# 3D Printed Organisms Enabled by Aspiration‐Assisted Adaptive Strategies

**DOI:** 10.1002/advs.202404617

**Published:** 2024-06-21

**Authors:** Guebum Han, Kanav Khosla, Kieran T. Smith, Daniel Wai Hou Ng, JiYong Lee, Xia Ouyang, John C. Bischof, Michael C. McAlpine

**Affiliations:** ^1^ Department of Mechanical Engineering University of Minnesota Minneapolis MN 55455 USA; ^2^ Center for Advanced Technologies for the Preservation of Biological Systems (ATP‐Bio) University of Minnesota Minneapolis MN 55455 USA; ^3^ Department of Fisheries Wildlife and Conservation Biology University of Minnesota Minneapolis MN 55108 USA; ^4^ Department of Biomedical Engineering University of Minnesota Minneapolis MN 55455 USA

**Keywords:** 3D printing, bionic organisms, cryopreservation, pick‐and‐place

## Abstract

Devising an approach to deterministically position organisms can impact various fields such as bioimaging, cybernetics, cryopreservation, and organism‐integrated devices. This requires continuously assessing the locations of randomly distributed organisms to collect and transfer them to target spaces without harm. Here, an aspiration‐assisted adaptive printing system is developed that tracks, harvests, and relocates living and moving organisms on target spaces via a pick‐and‐place mechanism that continuously adapts to updated visual and spatial information about the organisms and target spaces. These adaptive printing strategies successfully positioned a single static organism, multiple organisms in droplets, and a single moving organism on target spaces. Their capabilities are exemplified by printing vitrification‐ready organisms in cryoprotectant droplets, sorting live organisms from dead ones, positioning organisms on curved surfaces, organizing organism‐powered displays, and integrating organisms with materials and devices in customizable shapes. These printing strategies can ultimately lead to autonomous biomanufacturing methods to evaluate and assemble organisms for a variety of single and multi‐organism‐based applications.

## Graphical abstract

An aspiration‐assisted adaptive printer that tracks, harvests, and relocates living and moving organisms is developed. It enables printing organisms in cryoprotectant droplets, sorting live and dead organisms, positioning organisms onto target and curved surfaces, assembling organism‐powered displays, and integrating organisms with materials and devices. These capabilities can lead to the evaluation and assembly of organisms for single and multi‐organism‐based applications.

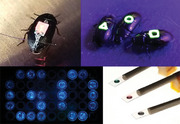

## 1 Introduction

The ability to manipulate and position organisms without compromising their integrity can find myriad applications. Zebrafish embryos,^[^ ^1^, ^2^, ^3^, ^4^, ^5^ ^]^ fruit fly embryos,^[^ ^6^, ^7^ ^]^ coral larvae,^[^ ^8^ ^]^ and beetles^[^ ^9^, ^10^ ^]^ have been cryopreserved for biodiversity preservation and aquaculture, serving as organism models for gaining insights into diseases, evolution, and ecology. Beetles^[^ ^11^, ^12^, ^13^ ^]^ and turtles^[^ ^14^ ^]^ have also been interfaced with devices to create cybernetic organisms for locomotive control, environmental monitoring, and behavioral sciences. Collections of organisms such as ant colonies,^[^ ^15^ ^]^ known as superorganisms, can yield an understanding of collective collaboration that can be utilized for distributed intelligence and multi‐robotic systems.^[^ ^16^, ^17^, ^18^, ^19^ ^]^ This requires tracking, collecting, and positioning randomly distributed organisms on target destinations, for applications such as cryogenic devices for vitrification,^[^ ^2^, ^6^ ^]^ soft gels for microinjection,^[^ ^20^ ^]^ bioimaging sample holders for monitoring,^[^ ^21^ ^]^ and task spaces for device integration.^[^ ^12^, ^22^ ^]^ These procedures have primarily relied on manual operation and handling, leading to limitations such as low‐throughput processes, inconsistencies, chemical safety issues, and contamination risks.^[^ ^23^, ^24^, ^25^ ^]^

Several approaches have been explored to position organisms. Microfluidic devices have been used to position zebrafish^[^ ^26^, ^27^ ^]^ and fruit fly embryos and larvae^[^ ^28^, ^29^ ^]^ using mechanical trapping structures in predetermined locations within the devices for culturing and monitoring. A vacuum‐based device has been developed to hold zebrafish embryos^[^ ^30^ ^]^ in prearranged suction apertures for microinjection. Robotic manipulators with angled micropipette tips have been employed to aspirate mammalian embryos^[^ ^31^ ^]^ into the tips and place them in a liquid medium for cryopreservation. Finally, a tube‐based system has been designed to transfer zebrafish larvae^[^ ^25^ ^]^ from reservoirs to a target location inside the tube for imaging. Despite this substantial progress, there remains a need to develop adaptive methodologies that can manage random tasks, including real‐time organism tracking and target space identification, with the objective of placing organisms via an automated system.

Extrusion‐based 3D printing systems have demonstrated the capabilities of manipulating biological materials and performing autonomous manufacturing. Line and dot printing methods have been used to position cells.^[^ ^32^, ^33^, ^34^ ^]^ Aspiration‐assisted printing methods, such as the Kenzan bioprinting method, have been used to pick, place, and assemble spheroids on needle arrays,^[^ ^35^, ^36^ ^]^ flat‐ended pin arrays,^[^ ^37^ ^]^ and self‐healing gels.^[^ ^38^, ^39^ ^]^ These methods have enabled the creation of scaffold‐based or scaffold‐free tissue models for regenerative medicine and drug screening. Computer‐vision tracking has allowed for autonomous printing processes that can deposit materials by adapting to changes in target surfaces, such as printing electronics on a moving hand,^[^ ^40^ ^]^ cell‐laden hydrogels on mice,^[^ ^40^ ^]^ and a strain sensor on an expanding lung.^[^ ^41^ ^]^ In principle, these capabilities can be extended to the autonomous tracking and positioning of organisms on target spaces. Yet, this application remains nascent and has primarily been explored for microorganisms, such as bacteria^[^ ^42^, ^43^, ^44^, ^45^ ^]^ and fungi,^[^ ^46^ ^]^ via simple line printing.

Here we introduce several aspiration‐assisted adaptive printing strategies to pick and place randomly distributed organisms in deterministic spaces. Aspiration‐assisted nozzles with and without mesh filters were fashioned to pick and place organisms without harm. Adaptive printing systems were also developed by integrating a multi‐nozzle printing system with machine vision and laser systems, enabling closed‐loop printing that adapts to updated visual and spatial information about organisms and target spaces. Utilizing the nozzles and adaptive systems, printing strategies were devised to track, pick, and place diverse organisms on target spaces: a single static zebrafish embryo, dinoflagellate‐, shrimp embryo‐, and shrimp larva–laden droplets, and a single moving beetle. The length scales and mechanical properties of these organisms are shown in Figure S1 (Supporting Information). The applications of these strategies were exemplified by generating vitrification‐ready droplets of zebrafish and shrimp embryos on arbitrarily‐placed cryotop devices, sorting live zebrafish embryos from dead ones, placing zebrafish embryos on curved surfaces, creating dinoflagellate‐based displays, and integrating beetles with symbols, electrodes, and light‐emitting diodes (LEDs) to create 3D printed bionic organisms.

## 2 Results and Discussion

### 2.1 Printing Strategy for a Single Static Organism

**Figure 1:**
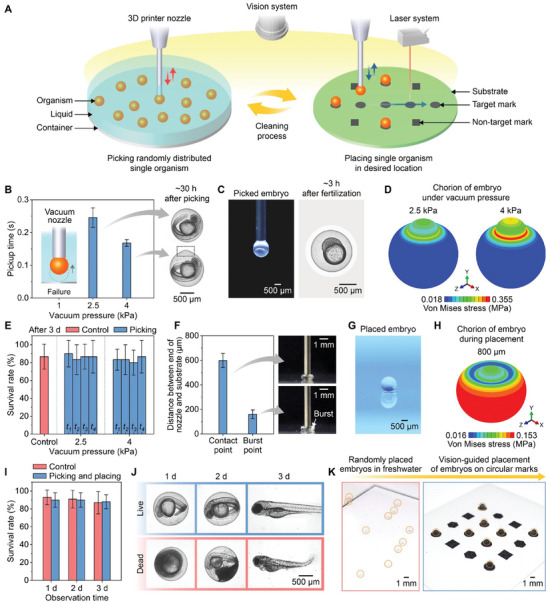
Aspiration‐assisted printing strategy for a single static organism. A) Schematic of the printing strategy. This strategy was developed using a testbed of zebrafish embryos. Characterization of the picking process for embryos: B) determination of optimal vacuum pressure (left, *n* = 15) based on the deformation of the chorions of the embryos (right) as shown by the dotted rectangle, C) embryo picked up by a nozzle under an optimal vacuum pressure of 2.5 kPa (left) and its observation after being separated from the nozzle (right), D) finite element (FE)‐predicted stress distribution of a chorion of an embryo during picking, and E) survival rates of embryos 3 d after the picking process (*t*_1_ = 0.5 s, *t*_2 _= 1 s, *t*_3_ = 2 s, and *t*_4_ = 3 s) (*n* = 30). Characterization of the placing process for embryos: F) determination of the nozzle distance from the substrate (left, *n* = 9), based on the critical burst distance as defined by the distance in which the blastoderm and yolk underwent structural damage due to compression by the nozzle (right), G) embryo placed with an optimal nozzle distance of 800 µm, and H) FE‐predicted stress distribution of the chorions of embryos during placing. I) Survival rates (*n* = 100) and J) images of embryos after the picking and placing processes. K) Embryos placed on circular target marks. The embryos were highlighted with dotted circles to assist in their visualization. The images in B), C) (right), and J) are optical microscope images, and the remainder of the images are photographs.

An aspiration‐assisted adaptive printing approach was developed to transfer a single static organism from a liquid to a target location (**Figure** **1**A). A printer first positions a nozzle on top of an organism by adapting to its updated 2D location from a machine vision system and picks it up by activating the vacuum pressure in the nozzle. The printer then transfers the organism to a target substrate via its 3D location as determined by machine vision and laser systems. Finally, the organism is placed by deactivating the vacuum pressure in the nozzle. Residual water accumulated inside the nozzle during picking is removed by alternating positive and vacuum pressures. This set of tasks required developing a printing system with incorporated machine vision and laser systems (Figure S2A, Supporting Information) and calibrating it with a testbed of zebrafish embryos, to adjust the printing parameters, maximize the survival rates of the printed embryos, and evaluate the adaptive printing performance.

This process was first investigated to collect zebrafish embryos in freshwater with the goal of minimal damage. A circular nozzle with a diameter of 510 µm was used to pick up a zebrafish embryo without allowing it to be siphoned into the nozzle under vacuum pressure. The nozzle was positioned on top of an embryo in a high cell stage in freshwater. Its end was 2 mm from the bottom of a reservoir (Figure S2B, Supporting Information). Under these conditions, the effect of vacuum pressure on the picking process of the zebrafish embryos was tested. While 1 kPa was insufficient, 2.5 and 4.0 kPa provided sufficient pressure to pick up embryos within 0.25 ± 0.03 s and 0.17 ± 0.01 s, respectively (Figure 1B). However, 4.0 kPa caused deformation of the chorions of the embryos as shown by the dotted rectangle in the image in Figure 1B, despite requiring less time to lift them. The variation in the pick time was likely due to differences in the embryo weights, as supported by their size variations (Figure S1B, Supporting Information). The representative images in Figure 1C show zebrafish embryos picked up at the optimal vacuum level of 2.5 kPa. Axisymmetric finite element (FE) models of the chorion and nozzle were developed to understand the origin of the deformation (Figure S3A, Supporting Information). The FE‐predicted stress distributions showed that the highly stressed region between the inner edge of the nozzle and the chorion caused deformation at 4 kPa (Figure 1D).

The effects of vacuum pressure and time on the survival rates of zebrafish embryos during the picking process were evaluated based on criteria adopted from previous studies (Table S1, Supporting Information).^[^ ^2^, ^47^ ^]^ Picking pressures of 2.5 and 4 kPa for all times yielded survival rates 3 d after picking of ca. 85%.  The experimental groups were not significantly different from the control group that did not undergo the picking process (*p *≥  0.71) (Figure 1E). However, deformation of the chorion was observed at 4 kPa irrespective of the vacuum times. These experimental and computational results concluded that the 510 µm diameter nozzle with 2.5 kPa of vacuum pressure for more than 0.25 s was optimal for picking up zebrafish embryos without damage.

A placing process was investigated to position zebrafish embryos on target locations without sacrificing their integrities. A 510 µm diameter nozzle picked up an embryo with the optimal vacuum pressure (2.5 kPa). Two quantitative measures, critical contact and burst distances, were determined by observing the embryo as it approached a rigid substrate, which consisted of a polypropylene (PP) film on a printer stage. As shown in Figure 1F, the critical contact distance was defined as the distance in which the end of the nozzle reached the blastoderm and yolk. The critical burst distance was defined as the distance in which the blastoderm and yolk underwent structural damage due to compression by the nozzle. The critical contact and burst distances were measured to be 598.44 ± 57.74 µm and 161.11 ± 35.00 µm from the substrate, respectively. Based on these results, the optimal nozzle distance for placing embryos was selected as 800 µm, greater than both critical distances. Figure 1G and Figure S4A (Supporting Information) show that embryos were placed at the optimal distance by deactivating the vacuum pressure. The residual water around the placed embryos was measured to be 0.08 ± 0.02 µL (Figure S4B, Supporting Information). The placing step was possible because the combined forces from gravity acting on an embryo, the adhesion between the embryo and the substrate inside the contact area, and the adhesion between the embryo and water outside the contact area were greater than the combined forces from the adhesion between the embryo and the nozzle and the adhesion between the embryo and water inside the nozzle. The FE‐predicted maximum stress of the chorion of an embryo at the optimal nozzle distance was ca. 0.15 MPa (Figure 1H; Figure S3B, Supporting Information), which was lower than the stress observed to cause the deformation of the chorion during picking (Figure 1D).

The survival rates of the zebrafish embryos were examined following aspiration‐assisted printing. Embryos in a high cell stage were picked and placed with the optimized conditions determined in the previous experiments (Figure 1B,F). The embryos were examined at various time points using a microscope to assess their morphological and developmental features, guided by survival criteria adopted from previous studies (Table S1, Supporting Information).^[^ ^2^, ^47^ ^]^ The survival rate of the experimental group was not significantly different from that of the control group, which was not subjected to the printing process (*p* ≥ 0.42) (Figure 1I). The representative images in Figure 1J visualize the distinct differences in features between live and dead embryos at the different observation times. Live embryos exhibited movements and heartbeats after 1 d, eye pigmentation after 2 d, swimming abilities, straight trunk musculature, and the presence of normal hearts after 3 d. In contrast, dead embryos showed disintegrated membranes after 1 d, lacked movement, heartbeats, or eye pigmentation after 2 d, and exhibited abnormal length, malformation of hearts and spines, or swimming inability after 3 d. These results indicated that the printing strategy was suitable for picking and placing embryos on target surfaces without harm.

After optimizing these aspiration‐assisted printing parameters, adaptive printing of zebrafish embryos was demonstrated by incorporating sensing of the embryos and target substrates as part of the printing process. One section of a dish (Figure S2B, Supporting Information) contained randomly distributed zebrafish embryos in freshwater, while the other was used to clean residual water in a nozzle. The adaptive printing system (Figure S2A, Supporting Information) with the nozzle picked up the embryos in freshwater based on their 2D locations acquired from the machine vision system (Figure S5A and Table S2, Supporting Information). This system differentiated circular target marks from square and hexagonal marks using the machine vision system (Figure S5B, Supporting Information). It then placed the embryos on the circular marks by measuring their 3D locations via the machine vision and laser systems. Finally, it removed residual water in the nozzle by alternating positive and vacuum pressures before entering the next printing cycle. Figure 1K and Movie S1 (Supporting Information) show this adaptive printing process. This process, with a smaller nozzle diameter than embryos, was also applicable to the concentrated population of embryos (Figure S5C,D, Supporting Information). The positioning error was estimated to be 128 ± 85 µm, primarily attributable to the rolling of embryos (Figure S6, Supporting Information). The vision system fails to track embryos when the embryos overlap beyond the 2D plane, and when the lighting condition is altered from the initial condition, because the filter parameters are not adaptive (Figure S5A, Supporting Information).

### 2.2 Printing Strategy for Multiple Organisms

**Figure 2:**
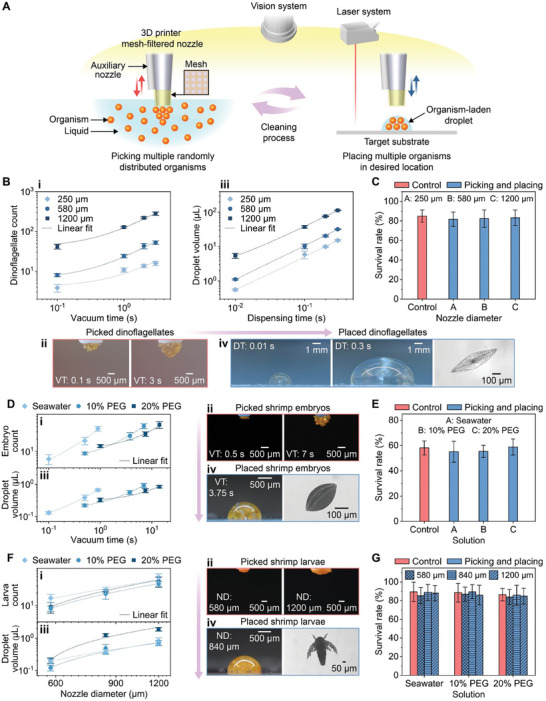
Aspiration‐assisted printing strategy for multiple organisms. A) Schematic of the printing strategy. This strategy was developed with testbeds of dinoflagellates, shrimp embryos, and shrimp larvae. B) Printing dinoflagellates in seawater droplets. i) Graph (*n* = 5) and ii) images (mesh‐filtered nozzle diameter (ND): 1200 µm) showing the effect of vacuum times (VTs) on the number of dinoflagellates picked at the end of a mesh‐filtered nozzle. iii) Graph (*n* = 5) and iv) images (left and center, mesh‐filtered ND: 1200 µm) showing the effect of dispensing times (DTs) on the volumes of placed dinoflagellate‐laden droplets. The rightmost image in (iv) shows a single dinoflagellate taken from the droplet. C) Survival rates of printed dinoflagellates (*n* = 15). D) Printing shrimp embryos in ≤ 1 µL droplets with different viscosities. i) Graph (*n* = 5) and ii) images (10% PEG) showing the effect of the VTs on the number of embryos picked at the end of the mesh‐filtered nozzle. iii) Graph (*n* = 5) showing the effect of the VTs on the volume of placed embryo–laden droplets. iv) Images showing an embryo–laden droplet (left, 10% PEG) and a single embryo taken from the droplet (right). E) Survival rates of printed shrimp embryos (*n* = 15). F) Printing shrimp larvae in ≤ 1 µL droplets with different viscosities. i) Graph (*n* = 5) and ii) images (10% PEG) showing the effect of the mesh‐filtered NDs on the number of picked larvae. iii) Graph (*n* = 5) showing the effect of the mesh‐filtered NDs on the volumes of placed larva–laden droplets. iv) Images showing a larva–laden droplet (left, 10% PEG) and a single larva taken from the droplet (right). G) Survival rates of printed shrimp larvae (*n* = 10). Each solution was tested with different NDs indicated by the fill patterns of the bar charts. The rightmost images in (B) iv), D) iv), and F) iv) are optical microscope images, while the remainder of the images are photographs.

An aspiration‐assisted adaptive printing method with a mesh‐filtered nozzle was devised to pick up multiple organisms in a liquid and place them on target locations (**Figure** **2**A). A mesh‐filtered nozzle was created by integrating a mesh filter with the end of a nozzle. The mesh filter allowed the nozzle to pick up organisms under vacuum pressure by separating them from a liquid, preventing the organisms from clogging the nozzle. An auxiliary nozzle was maintained under vacuum pressure during the stage movement following the picking process to aspirate any undesired liquid drops from the side of the mesh‐filtered nozzle. The mesh‐filtered nozzle placed the organisms on a target location by dispensing the liquid inside the nozzle under positive pressure, resulting in an organism‐laden droplet. The residual liquid in the mesh‐filtered nozzle was removed by alternating positive and vacuum pressures at the cleaning area. This printing process can adapt to real‐time visual and spatial information about a sample reservoir or target substrates obtained from the machine vision and laser systems. This adaptive printing system (Figure S2A, Supporting Information) was designed and tested with dinoflagellates, brine shrimp embryos, and brine shrimp larvae.

The picking process of dinoflagellates using mesh‐filtered nozzles was investigated. Based on preliminary experiments (Figure S7 and Table S3, Supporting Information), a nylon filter with a mesh size of 18 µm was selected and integrated with nozzles of various diameters (250, 580, and 1200 µm) to screen dinoflagellates from seawater. A 1.71% w/v solution of dinoflagellates in seawater was distributed within the reservoir by using compressed air from a nozzle (Figure S2C, Supporting Information). Mesh‐filtered nozzles were then immersed in the solution to pick up dinoflagellates at their ends under vacuum pressure at different times (2.5 kPa for 0.1, 1, 2, and 3 s). The number of picked dinoflagellates was counted as described in Figure S8 (Supporting Information). It increased with the vacuum time for all nozzle diameters (Figure 2B‐i,ii) (positive linear correlation: R^2^ > 0.93). This demonstrated the ability of mesh‐filtered nozzles to control the number of picked dinoflagellates by adjusting the vacuum time. The variation in the picked dinoflagellate counts may stem from differences in weights and sizes (Figure S1B, Supporting Information), potentially impacting the accumulation configurations at the nozzle ends.

Next, the placing process of dinoflagellates was examined. The mesh‐filtered nozzles were used to pick up dinoflagellates in seawater under vacuum pressure (2.5 kPa for 3 s) and place dinoflagellate‐laden seawater droplets ca. 350 µm away from a PP substrate by adjusting the dispensing time of positive pressure (0.7 psi for 0.01, 0.1, 0.2, and 0.3 s). The total droplet volume increased as the dispensing time increased for all nozzle diameters (Figure 2B‐iii,iv) (positive linear correlation: *R* ^2^ > 0.99). The total droplet volume also increased with increasing nozzle diameter. The formation of dinoflagellate‐laden droplets was likely driven by the positive pressure, the self‐weight of the droplets, and the adhesion between the droplets and substrate.^[^ ^48^, ^49^ ^]^ These results confirmed that mesh‐filtered nozzles could control the total volumes of dinoflagellate‐laden droplets by changing the dispensing times and nozzle diameters.

The survival rates of dinoflagellates were evaluated after the printing process. The mesh‐filtered nozzles picked and placed dinoflagellates using the longest vacuum and dispensing times from the previous experiments (Figure 2B‐i,iii). Dead dinoflagellates were stained with a trypan blue solution (Figure S9, Supporting Information). The results showed that the printing process did not significantly affect the survival rates of dinoflagellates compared to a control group that did not undergo any manipulation process (*p* ≥  0.24) (Figure 2C). Therefore, the mesh‐filtered nozzles can successfully pick and place dinoflagellates in seawater droplets without damage.

Next, the aspiration‐assisted printing strategy with mesh‐filtered nozzles was investigated to create microscale (≤ 1 µL) shrimp embryo–laden droplets of varying viscosities. Reservoirs containing 3.42% w/v solutions of shrimp embryos in seawater, 10% w/w polyethylene glycol (PEG), and 20% w/w PEG were prepared. Adding PEG to seawater increased the viscosity of the solution, as shown in Figure S10 (Supporting Information). Based on preliminary experiments (Figure S7 and Table S3, Supporting Information), the mesh‐filtered nozzle for seawater droplets had a diameter of 250 µm and a mesh size of 18 µm, while the nozzle for 10% w/w and 20% w/w PEG droplets had a diameter of 410 µm and a mesh size of 60 µm. The larger nozzle diameter and mesh size were used for 10% w/w PEG and 20% w/w PEG, considering their higher viscosities than seawater. The solutions were stirred with compressed air (Figure S2C, Supporting Information). The mesh‐filtered nozzles picked up shrimp embryos at different vacuum times (seawater: 4 kPa for 0.1, 0.5, and 0.9 s; 10% w/w PEG: 2 kPa for 0.5, 3.75, and 7 s; and 20% w/w PEG: 4 kPa for 1 s, 7.5, and 14 s). The mesh‐filtered nozzles placed shrimp embryo–laden droplets ca. 350 µm away from a PP substrate by applying positive pressure (seawater: 0.7 psi for 0.03 s, 10% w/w PEG: 1 psi for 0.03 s, and 20% w/w PEG: 1.3 psi for 0.03 s). The number of picked shrimp embryos (Figure S8, Supporting Information) and the volumes of shrimp embryo–laden droplets were measured.

As the vacuum time increased, the mesh‐filtered nozzles picked up larger numbers of shrimp embryos in seawater, 10% w/w PEG, and 20% w/w PEG (Figure 2D‐i,ii) (positive linear correlation: *R* ^2^ > 0.98). Furthermore, an increase in vacuum time led to a larger droplet volume for all solutions (Figure 2D‐iii,iv) (positive linear correlation: *R* ^2^ > 0.99). These results demonstrated that this printing method could generate microscale (≤ 1 µL) shrimp embryo–laden seawater and PEG droplets by adjusting the vacuum time. The variations in the counts of the picked shrimp embryos could be attributed to the variations in their weights and sizes (Figure S1B, Supporting Information), possibly affecting their accumulation patterns at the nozzle ends.

The survival rates of shrimp embryos after printing were tested. Shrimp embryo–laden droplets of seawater, 10% w/w PEG, and 20% w/w PEG were printed using the longest vacuum time applied for each solution in Figure 2D. Shrimp embryos were considered alive if they hatched and swam after 1 d of incubation in seawater after printing. For all solutions, the survival rates of the printed shrimp embryos were not significantly different from those of a control group that did not undergo the printing process (*p* ≥ 0.11) (Figure 2E). Therefore, the mesh‐filtered nozzles could generate shrimp embryo–laden seawater and PEG droplets without harming the embryos.

The aspiration‐assisted printing approach with mesh‐filtered nozzles was examined to generate microscale (≤ 1 µL) shrimp larva–laden droplets with different viscosities. Solutions of shrimp larvae in seawater, 10% w/w PEG, and 20% w/w PEG were prepared at a concentration of 2.6% w/v. The viscosity of the solution increased when PEG was added to seawater, as shown in Figure S10 (Supporting Information). Based on preliminary results (Figure S7 and Table S3, Supporting Information), nozzles with diameters of 580, 840, and 1200 µm were integrated with filters of 18 µm mesh size for seawater and 60 µm mesh size for 10% and 20% w/w PEG solutions. The nozzle diameter was an independent variable to control the number of picked shrimp larvae. The solutions were stirred using compressed air from the nozzle for distribution (Figure S2C, Supporting Information), and then the mesh‐filtered nozzles picked up shrimp larvae under vacuum pressure (seawater: 4 kPa for 0.2 s, 10% w/w PEG: 2 kPa for 1 s, and 20% w/w PEG: 4 kPa for 2 s). The nozzles placed shrimp larva–laden seawater and PEG droplets ca. 350 µm away from a PP substrate with positive pressure (seawater: 0.7 psi for 0.03 s, 10% w/w PEG: 1 psi for 0.03 s, and 20% w/w PEG: 1.3 psi for 0.03 s). The number of picked shrimp larvae (Figure S8, Supporting Information) and the volumes of shrimp larva–laden droplets were evaluated.

As the nozzle diameter increased, the number of shrimp larvae picked from seawater, 10% w/w PEG, and 20% w/w PEG increased (Figure 2F‐i,ii) (positive linear correlation: *R* ^2^ > 0.93). In addition, increasing the nozzle diameter resulted in a larger volume of the shrimp larva–laden droplet for all solutions (Figure 2F‐iii,iv) (positive linear correlation: *R* ^2^ > 0.96). These findings demonstrated that the mesh‐filtered nozzle could generate microscale (≤ 1 µL) shrimp larva–laden seawater and PEG droplets by tuning the nozzle diameter. They also suggest that the nozzle diameter can control the number of picked organisms in addition to the vacuum time (Figure S11, Supporting Information). The variation in the number of the picked shrimp larvae may be due to differences in their sizes (Figure S1B, Supporting Information), weights, and movement capabilities, which could influence their collection and accumulation at the ends of the nozzles.

The survival rates of shrimp larvae after printing were investigated. Shrimp larva–laden droplets were printed with seawater, 10% w/w PEG, and 20% w/w PEG using the same conditions as shown in Figure 2F. Shrimp larvae were incubated in seawater for 1 d after printing and considered to be alive if they swam. For all solutions, the survival rates of the printed shrimp larvae exhibited no significant differences when compared to a control group that did not undergo the printing process (*p* ≥ 0.3) (Figure 2G). This showed that the mesh‐filtered nozzles could create shrimp larva–laden seawater and PEG droplets without damage.

The printing strategy for droplets laden with dinoflagellates, shrimp embryos, and shrimp larvae can operate in a closed‐loop fashion by adapting to the updated locations of sample reservoirs or target substrates via the machine vision and laser systems, similar to the adaptive printing approach used for zebrafish embryos. This is highlighted in Movie S2 (Supporting Information), which demonstrates the placement of a shrimp embryo–laden droplet on a randomly positioned target substrate through the adaptive adjustment to its location. The positioning error of the strategy was 64 ± 26 µm, estimated by autonomously placing a shrimp embryo–laden droplet on a circular target mark (Figure S12, Supporting Information).

### 2.3 Printing Strategy for a Single Moving Organism

**Figure 3:**
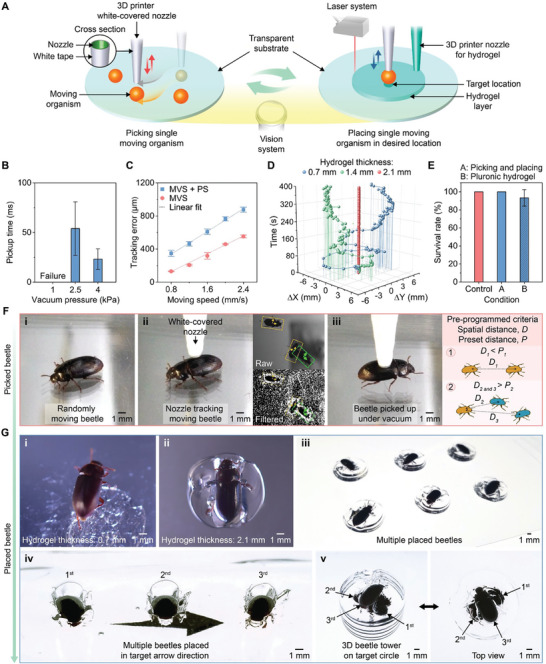
Aspiration‐assisted printing strategy for a single moving organism. A) Schematic of the printing strategy. This strategy was realized with a testbed of beetles. Characterization of the picking process for beetles: B) determination of the vacuum pressure (*n* = 5) and C) tracking errors of the adaptive printing system (MVS: machine vision system and PS: printing system) (*n* = 5). Characterization of the placing process for beetles: D) trajectories of the beetles in different Pluronic hydrogel thicknesses. E) Survival rates of the beetles after printing and removing Pluronic hydrogel (*n* = 30). F) Beetles tracked and picked up by a white‐covered nozzle: i) a randomly moving beetle on a transparent substrate, ii) a nozzle tracking the beetle (left) based on its spatial location as detected by the machine vision system (right), and iii) a beetle picked up by a nozzle (left) when it meets pre‐programmed criteria (right). G) Beetles placed in Pluronic hydrogel by a white‐covered nozzle: i) a beetle escaping from a thin hydrogel structure, ii) a beetle placed in a thick hydrogel structure, iii) an array of beetles created via immobilization in thick hydrogel structures, iv) beetles placed along with a target arrow direction in a hydrogel bath, and v) a beetle tower created with hydrogel on a target circle. The target circle, which is covered by the beetles, can be observed in Movie S4 (Supporting Information). The images in F and G are photographs.

An aspiration‐assisted adaptive printing strategy was designed to track, pick, and place a single moving organism on a target space (**Figure** **3**A). A vision camera was placed underneath a transparent substrate to perform real‐time imaging of moving organisms and target locations. To reduce background noise and enhance the contrast between the background and organisms that are non‐white or non‐transparent in imaging, a nozzle and its mechanical support that tracks organisms were covered in white color, and a white board was placed above them (Figure S13, Supporting Information). The machine vision system acquired visual and spatial (2D location) information about the organisms and target locations by analyzing the imaging results. The laser system acquired the heights of the target locations. A printing system with a white‐covered nozzle tracked and picked up a moving organism under vacuum pressure by adapting to its location. The printing system with another nozzle printed a hydrogel structure on a target surface and placed the organism in the hydrogel by deactivating the vacuum pressure. To realize this strategy, an adaptive printing system (Figure S13, Supporting Information) was developed and examined with a testbed of beetles. This included demonstrating the adaptive printing capability, investigating the printing parameters, and assessing the survival rates of the beetles after printing.

The vacuum pressures to pick up the beetles were investigated. A 1200 µm diameter nozzle under different vacuum pressures (1, 2.5, and 4 kPa) was positioned ca. 1 mm above the top of a beetle that was immobilized by ethanol. 1 kPa was insufficient to pick up a beetle, while 2.5 and 4 kPa were sufficient (Figure 3B). 4 kPa required 23.2 ± 10.33 ms to pick up a beetle, which was approximately half as much time as 2.5 kPa. The discrepancy was likely due to the differences in weight among the beetles, as supported by their size variations (Figure S1B, Supporting Information). No notable traces were observed at the locations where the nozzle made contact with the beetles, as beetles possess higher elastic moduli (Figure S1, Supporting Information) than the applied vacuum pressures, such that it is unlikely that they would undergo significant deformation. In conclusion, 4 kPa was selected as the optimal vacuum pressure to pick up the beetles.

The tracking performance of a machine vision‐enabled adaptive printing system for a moving organism was evaluated. A motorized stage moved a circular target at various speeds (Figure S14, Supporting Information). A 200 µm diameter nozzle with black‐colored Pluronic hydrogel tracked the circular target ca. 1 mm above its top surface and printed two dots on the target. The first dot was printed before the target began moving, and the second dot was printed while the target was in motion. The distance between the two dots was measured to assess the combined tracking error of the machine vision and printing systems. Furthermore, the displacement of the moving target between successive vision detections was measured to quantify the tracking error attributed to the machine vision system. Both tracking errors increased as the speed of the target increased (Figure 3C) (positive linear correlation: *R* ^2^ > 0.99). The primary contributors to the combined tracking error were the tracking error of the machine vision system and the delay caused by the nozzle approaching the moving target. These findings suggested that if the millimeter‐scale beetle (Figure S1, Supporting Information) moved faster than a speed threshold, then the adaptive printing system could not successfully pick it up due to the tracking error. This emphasized the need to include a filtering algorithm in the printing process that allowed the nozzle to pick up a beetle only when moved more slowly than a speed threshold, as described below.

Hydrogel structures were used to confine beetles to target locations. A 3 mm diameter nozzle was used to print 40% w/v Pluronic hydrogel into cylindrical structures with a diameter of ca. 10 mm and thicknesses of 0.7, 1.4, and 2.1 mm. A beetle was picked up using the 1200 µm diameter nozzle under vacuum pressure and then placed in the hydrogel structure by deactivating the vacuum pressure when the nozzle was positioned ca. 1.5 mm above the substrate. The machine vision system was used to track the placed beetle and measure the time required for the beetle to move a displacement of at least 500 µm from its initial position. The result showed that the beetles placed in hydrogel structures with thicknesses of 0.7, 1.4, and 2.1 mm required times of 29.33 ± 6.11 s, 44.00 ± 4.00 s, and 637.33 ± 59.37 s, respectively (*n* = 5). Figure 3D shows the representative trajectories of beetles placed in the different hydrogel thicknesses. These findings demonstrated that the thickness of the hydrogel structure can be used to control the confinement time of the beetles after placing.

The impact of the printing process on the survival of the beetles was assessed. Using the aspiration‐assisted nozzle with the optimized vacuum condition (Figure 3B), the first group of beetles was picked and placed into a petri dish. The second group of beetles was picked up, placed in 2.1 mm thick Pluronic hydrogel structures, and removed from the hydrogel structures after 10 min using tweezers and water. The survival rates of the two groups were measured 1 d after the experiments. The results showed that the pick‐and‐place process had no impact on the survival rate of the beetles in comparison to a control group that did not undergo the process (*p* = 1) (Figure 3E). Following the removal of the Pluronic hydrogel, the survival rate of the beetles decreased to 93.33 ± 9.13%. However, this decrease was not statistically significant compared to the control group (*p* = 0.44). Therefore, it was concluded that the printing strategy could be used to pick and place beetles onto target locations without harm.

After investigating each printing step, adaptive printing for beetles was demonstrated by using their visual and spatial information as a component of the printing process. For the picking process, a freely moving beetle was placed on a transparent substrate (Figure 3F‐i). The printing system with the 1200 µm diameter white‐covered nozzle tracked the beetle by adapting to its real‐time location, as sensed by the machine vision system using image processing filters (Figure 3F‐ii; Figure S15A, Supporting Information). The system used an algorithm to pick up a beetle via the nozzle under vacuum pressure only when it met two criteria calculated using its spatial information (Figure 3F‐iii). The first criterion was that the difference in the distance between consecutive vision detections of the beetle had to be less than a preset distance of 400 µm, which was related to the speed of the moving beetle, as shown in Figure 3C. It ensured that the nozzle picked up the beetle only when it was moving at a sufficiently slow speed. When tracking multiple beetles, the second criterion required the target beetle to be isolated from other beetles by at least a preset distance of 7.5 mm. It ensured that the nozzle picked up the target beetle without its legs becoming entangled with other beetles. The demonstration of tracking and picking beetles through this adaptive printing system using these two criteria is shown in Movie S3 (Supporting Information). The second criterion was demonstrated using two beetles, one alive and one dead, with a preset distance of more than 15 mm for improved visualization.

After tracking and picking the beetles, the adaptive printing system placed them on various target locations using Pluronic hydrogel. First, the printing system created hydrogel structures with 0.7 and 2.1 mm thicknesses and placed beetles in these structures (Figure 3G‐i,ii). As evidenced in Figure 3D, the thicker hydrogel structure prevented the beetle from escaping for a longer duration than the thin hydrogel structure. Utilizing the thicker hydrogel structure, multiple beetles were placed at a horizontal interval of ca. 20 mm (Figure 3G‐iii). Second, the machine vision system measured the 2D location and angle of a target arrow pattern on the bottom of a Pluronic hydrogel bath (Figure S15B, Supporting Information). Based on the spatial information of the pattern, the printing system placed multiple beetles in the hydrogel bath (Figure 3G‐iv). Third, the machine vision and laser systems detected the 3D location of a circular target mark (Figure S15C, Supporting Information). The printing system then stacked multiple beetles by constructing a Pluronic hydrogel structure on the target mark (Figure 3G‐v). The printing processes of these examples can be found in Movie S4 (Supporting Information). The positioning error of the printing strategy for beetles was estimated to be 171 ± 80 µm (Figure S16, Supporting Information). These results demonstrated the capability of this strategy to track, pick, and place freely moving beetles in deterministic locations.

### 2.4 Applications of Printing Organisms

#### 2.4.1 High‐Throughput Cryopreservation of Zebrafish Embryos

**Figure 4:**
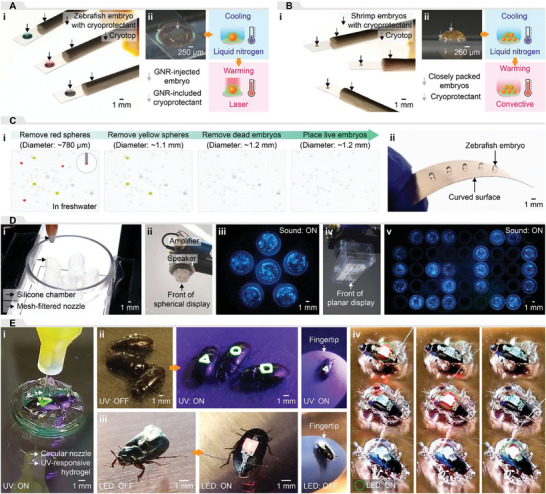
Applications of aspiration‐assisted printing of organisms. A) Zebrafish embryo‐included cryoprotectant droplets: i) embryos with different cryoprotectant droplets placed on cryotop devices at random locations and heights, and ii) cryopreservation and laser rewarming of gold nanorod (GNR)‐injected embryos with GNR‐included cryoprotectant droplets. B) Shrimp embryo–laden cryoprotectant droplets: i) embryos with cryoprotectant droplets placed on cryotop devices at random locations and heights, and ii) their cryopreservation and convective rewarming. C) Sorting and conformal placing of live zebrafish embryos: i) the process of sorting live embryos from dead embryos and microspheres, and ii) placing live embryos on a curved copper surface. The inset in (i) shows an example of picking up a microsphere via aspiration‐assisted printing. D) Dinoflagellate‐based displays: i) printing dinoflagellates into silicone chambers that were conformally printed on a spherical surface, ii) spherical display attached to a speaker for testing, iii) spherical display emitting bioluminescence in response to audio‐induced vibrations, iv) planar display attached to a speaker for testing, v) planar display displaying the letters “GO” in response to audio‐induced vibrations. E) Beetles with integrated secret symbols and electronic devices: i) printing UV‐responsive secret symbols (3 layers) on a beetle placed in a hydrogel structure, ii) beetles displaying secret symbols (triangle, square, and circle) under UV light, and their scale relative to a fingertip, iii) beetle with integrated electrodes and light‐emitting diode (LED), and its scale relative to a fingertip, and iv) multiple beetles with integrated electrodes and LEDs in a hydrogel bath. All images are photographs.

Next, we explored diverse applications to exemplify the capabilities of the strategies developed for printing organisms. First, we showed whether the use of the adaptive printing strategy described above (Figure 1) for a single static organism could be used to place zebrafish embryos and cryoprotectant droplets on cryotop devices autonomously. The machine vision system measured the 2D locations of embryos and cryotop devices (Figure S17A, Supporting Information). The laser system measured the heights of the cryotop devices based on their locations from the machine vision system. By adapting to the spatial information, the multi‐nozzle printer picked up the randomly distributed embryos in freshwater using a 510 µm diameter nozzle, placed them on cryotop devices which were located at random locations and heights, and created viscous cryoprotectant droplets (ca. 3 µL) of different colors to immerse the embryos using a 150 µm diameter nozzle (**Figure** **4**A‐i; Figure S10 and Movie S5, Supporting Information). The colored droplets were formulated by dispensing the cryoprotectant agents mixed with green and red dyes, either separately or together.

This strategy was applied to improve the throughput of a cryopreservation method for zebrafish embryos which was previously developed through manual manipulation.^[^ ^1^, ^2^ ^]^ A cryoprotectant agent with gold nanorods (GNRs) was microinjected into the yolks of embryos. The adaptive printing system placed the microinjected embryos and GNR‐included cryoprotectant droplets on cryotop devices (Figure 4A‐ii). The embryos were rapidly placed in liquid nitrogen for vitrification, then removed from the liquid nitrogen and rewarmed using a laser. The printing system prepared a vitrification‐ready embryo droplet on a cryotop device within ca. 13 s, which was ≈12 times faster than the manual method which requires 150 s.^[^ ^1^, ^2^ ^]^ The manual approach used pipettes and tissue paper to pick up, place, and clean embryos and create cryoprotectant droplets. The breakdown of the times required for both methods can be found in the Supporting Information. The survival rates of placed embryos after cryopreservation and rewarming were consistent with previous studies^[^ ^1^, ^2^ ^]^ (Figure S17B and Movie S5, Supporting Information). These results showed that the printing approach could enhance the cryopreservation throughput of zebrafish embryos compared to manual manipulation while maintaining survival rates.

#### 2.4.2 High‐Throughput Cryopreservation Of Shrimp Embryos

The adaptive printing approach for multiple organisms (Figure 2) was utilized to place shrimp embryo–laden cryoprotectant droplets on cryotop devices. A 7.33% w/v solution of shrimp embryos in a viscous cryoprotectant agent (Figure S10, Supporting Information) was prepared in a reservoir. The machine vision system sensed the 2D locations of the reservoir and cryotop devices (Figure S17A, Supporting Information). In addition, the machine vision‐guided laser system measured the heights of the cryotop devices. Using the spatial data, the adaptive printing system with a mesh‐filtered nozzle (diameter: 250 µm and mesh size: 60 µm) picked up embryos in the reservoir and placed embryo–laden cryoprotectant droplets on the cryotop devices (Figure 4B‐i; Movie S5, Supporting Information). The embryos were quickly immersed in liquid nitrogen for vitrification and convectively rewarmed in seawater at room temperature (Figure 4B‐ii).

The adaptive printing method placed the shrimp embryo–laden cryoprotectant droplet on the cryotop device in ca. 20 s. The droplet had a volume of 0.45 ± 0.09 µL and contained 32.50 ± 5.85 embryos. Compared to the manual method, this time was shorter, and the number was higher. The manual method required 150 s to create a shrimp‐laden cryoprotectant droplet with a volume of 1 µL containing ca. 10 embryos. It included tasks such as collecting, positioning, and cleaning embryos, and dispensing cryoprotectant droplets using pipettes and tissue paper. A breakdown of the times required for both methods is provided in the Supporting Information. The survival rate of cryopreserved and rewarmed embryos after printing was not significantly different from the control group (Figure S17C; Movie S5, Supporting Information). These results demonstrated that the printing strategy could increase the cryopreservation throughput of shrimp embryos by ca. 24× compared to manual manipulation via a combination of reducing the droplet creation time and increasing the number of embryos per droplet.

While the demonstration of the printing methodologies showed the potential to enhance organism cryopreservation, additional advancements are necessary to achieve the desired throughputs. We anticipate that cryopreserving organisms for specific genetic lines and aquaculture breeding programs may require a throughput of 10000 embryos per day. Similarly, for food production purposes, a cryopreservation throughput of 100000 embryos per day would likely be required. These estimated throughputs would depend on both the survival rates of the organism cryopreservation process and the specific organisms.

#### 2.4.3 Sorting and Conformal Printing of Live Zebrafish Embryos

Live zebrafish embryos were sorted via the adaptive printing strategy described above (Figure 1). A petri dish filled with freshwater contained live zebrafish embryos and undesired objects such as dead zebrafish embryos and red and yellow microspheres. The machine vision system differentiated live embryos from other objects by adjusting the contrast conversion filter (Figure S18, Supporting Information). The 2D locations of all objects were then measured, and the printing system with a 510 µm diameter nozzle sorted live embryos by picking and relocating undesired objects to a position outside the petri dish (Figure 4C‐i; Movie S6, Supporting Information). If the target objects overlap with embryos or other objects to the extent that they no longer exhibit similarity to their reference image for the pattern search (Figure S18 and Table S2, Supporting Information), the vision system will fail to distinguish them. Additionally, tracking failure can occur when the lighting conditions deviate from the initial settings, as the filter parameters are non‐adaptive (Figure S18, Supporting Information).

The strategy was also applied to conformally place live zebrafish embryos on a curved surface. The machine vision system detected the 2D locations of live embryos in freshwater. It also measured the 2D location of a curved copper plate (curvature: 0.35) and guided the laser system to measure the height of the plate at five different locations with a 4 mm interval (Figure S19 Supporting Information). Using the spatial information, the printing system with a 510 µm diameter nozzle placed live zebrafish embryos on the curved plate (Figure 4C‐ii; Movie S6, Supporting Information). These examples showed that the adaptive printing strategy could distinguish live zebrafish embryos from other objects and conformally place them on curved target surfaces.

#### 2.4.4 Dinoflagellate‐Based Spherical and Planar Displays

The mesh‐filtered nozzle printing strategy (Figure 2) was employed to fabricate dinoflagellate‐based spherical displays that exhibited bioluminescence in response to mechanical vibrations. The printing system with a 330 µm diameter nozzle was utilized to conformally print six chambers (radius: 3 mm and height: 6.25 mm) on the inner surface of a hemispherical glass dome (diameter: ca. 15 mm) with room temperature vulcanizing (RTV) silicone. The printing system with a mesh‐filtered nozzle (diameter: 1200 µm and mesh size: 18 µm) picked up dinoflagellates in seawater (2.32% w/v) from the reservoir and placed dinoflagellate‐laden seawater droplets into the chambers using their locations as detected by the machine vision system (Figure 4D‐i). The detailed fabrication process is provided in Figure S20A (Supporting Information). The dinoflagellate‐based spherical display was attached to a speaker (Figure 4D‐ii) for testing. The chambers of the spherical display, functioning as dinoflagellate‐powered pixels, emitted bioluminescence in response to vibrations induced by the speaker sound (Figure 4D‐iii; Figure S20B, and Movie S7, Supporting Information).

The printing strategy was then utilized to fabricate dinoflagellate‐based planar displays. A 5 × 9 array of RTV silicone chambers (radius: 3 mm, height: 6.5 mm, and interval: 7.5 mm) was printed on a polyethylene terephthalate (PET) film. The letters “GO” were drawn by coloring the PET film on the opposite side of the chambers. The machine vision system detected the locations of the colored chambers. The printing system with the mesh‐filtered nozzle placed dinoflagellate‐laden seawater droplets in the chambers. This fabrication process is illustrated in Figure S20C (Supporting Information). The planar display was attached to a transparent container fixed to a speaker (Figure 4D‐iv). In response to audio‐induced vibrations, the chambers of the planar display exhibited bioluminescence, displaying the letters “GO” (Figure 4D‐v, Figure S20D, and Movie S7, Supporting Information).

#### 2.4.5 Beetles with Integrated Symbols and LEDs

Using the adaptive printing method for a moving organism (Figure 3), beetles were interfaced with customizable “secret” symbols. The machine vision‐guided adaptive printer with a white‐covered nozzle (diameter: 1200 µm) tracked, picked, and placed a moving beetle with a Pluronic hydrogel structure on a target location. Subsequently, the machine vision system detected the 2D location and angle of the placed beetle and guided the laser system to project a customizable 2D symbol profile onto its surface. Utilizing the projected profile, the printing system with a 100 µm diameter nozzle conformally printed a three‐layered symbol on the beetle (Figure 4E‐i). The symbol was created using a mixture of Pluronic hydrogel and ultraviolet (UV) printer ink. The ink remained invisible without UV light. Lastly, the beetle was released from the hydrogel structure used for confinement. The detailed printing process is provided in Figure S21 and Movie S8 (Supporting Information). Figure 4E‐ii shows beetles displaying secret symbols (triangle, circle, and square) when exposed to UV light. It also shows the relative scale between the beetle with the integrated symbol and a human fingertip.

The adaptive printing method was then utilized to integrate electrodes and LEDs with beetles. The machine vision‐guided adaptive printing system placed a beetle in a Pluronic hydrogel structure and acquired its 2D location and angle. The spatial information was used to guide the laser system to project a 2D electrode profile on the surface of the beetle (Figure S21B, Supporting Information). The printing system with a 150 µm diameter nozzle conformally printed the top and bottom electrodes on the beetle using the projected profile and a silver epoxy adhesive. A surface‐mounted LED, a representative example of a discrete electronic component, was aligned with the angle of the beetle using its spatial information. It was then automatically integrated with the printed electrodes via a pick‐and‐place method using a 510 µm diameter nozzle. Figure 4E‐iii shows the beetle with the integrated electrodes and LED after removing the Pluronic hydrogel structure which held the beetle in place, and the relative scale between the beetle and a human fingertip. Figure 4E‐iv shows multiple beetles with integrated electrodes and LEDs placed in a Pluronic hydrogel bath (Figure S22, Supporting Information). The functionality of the integrated electrodes and LEDs was confirmed by turning on the LEDs using high‐frequency electromagnetic field‐based wireless power transmission from a Tesla coil. A short length of wire contacted one side of the electrode to improve the wireless power transmission. As shown in Figure S23 (Supporting Information), the wire can be removed by optimizing the printed electrode design, thereby improving the efficiency of the wireless power transmission. The printing process and functional testing are presented in Movie S9 (Supporting Information). These demonstrations showed that the adaptive printing method can enable the integration of printable materials and electronic components with living organisms such as beetles.

## 3 Conclusion

We introduced aspiration‐assisted adaptive printing strategies that involve picking and placing randomly distributed organisms on target spaces. The aspiration‐assisted nozzles with and without mesh filters could manipulate organisms without harm. The printing system was integrated with machine vision and laser systems that adapted in real time to updated visual and spatial information about the organisms and target spaces. With the adaptive printing system, the nozzle picked and placed randomly distributed zebrafish embryos on target marks by differentiating them from other marks. The mesh‐filtered nozzle created dinoflagellate‐, shrimp embryo‐, and shrimp larva–laden droplets on target surfaces. The nozzle tracked and picked freely moving beetles when they met pre‐programmed criteria regarding their speeds and distances from other beetles. It then placed and assembled them in hydrogel structures that were printed on target spaces. These successful outcomes demonstrated the ability of these adaptive printing methods to manipulate organisms. These methods could replace the manual handling of organisms, reducing inconsistencies and contamination risks, while increasing the throughput. Furthermore, we envision their applicability to various organism types by adjusting printer nozzle diameters, mesh sizes, and vacuum pressure levels. After adjusting the parameters, the critical criterion for acceptability would be whether organisms can be picked up under vacuum pressure without being sucked into the nozzle or experiencing a decrease in the survival rate. If target organisms have differences in sizes and weights, the current strategies may not be able to pick them up without adjusting vacuum levels and nozzle sizes accordingly.

Our adaptive printing strategies for organisms have a wide range of applications. They permitted the autonomous placement of vitrification‐ready cryoprotectant droplets of zebrafish and shrimp embryos on randomly located cryotop devices. They also enabled the sorting of live zebrafish embryos from dead ones and their conformal placement on curved surfaces. Furthermore, they were applied to place dinoflagellates in silicone chambers that were conformally constructed on target surfaces, resulting in dinoflagellate‐based displays. These methods facilitated the interfacing of secret symbols and electronic materials in customizable shapes on beetles. These adaptive printing capabilities can enhance the cryopreservation throughput of organisms,^[^ ^2^, ^6^, ^8^ ^]^ create arrays of organisms with and without hydrogel encapsulation for bioimaging and microinjection techniques,^[^ ^21^, ^27^, ^50^ ^]^ and assemble remotely controlled cybernetic organisms with biointerfaced devices.^[^ ^12^, ^22^ ^]^ Ultimately, this ability to manipulate organisms can offer insight into collective behavior, which can be leveraged for engineering applications such as multi‐robot systems or distributed intelligence,^[^ ^16^, ^17^, ^18^, ^19^ ^]^ and potentially allow for the automated assembly of complex arrangements of organisms into superorganism hierarchies for harvesting, replication, and managed release.

Future studies will focus on the advancement of aspiration‐assisted adaptive printing strategies for organisms, with an emphasis on the following areas: i) incorporating 3D computer‐vision tracking to assess, pick, and place organisms that move in 3D spaces beyond the 2D plane, ii) integrating extrusion head‐mounted machine vision cameras with mobile robotic platforms to collect and assemble organisms over large areas and in complex environments, iii) developing adaptive printer nozzles that adjust the vacuum pressure based on the organism size to pick up specimens of varying sizes without the need to change nozzles, iv) integrating pressure sensors into printer nozzles for closed‐loop control in collecting multiple organisms with improved consistencies, v) developing active techniques for capturing, holding and releasing collections of different organisms for distributed information gathering and fetching, and vi) applying adaptive printing methods to collect social insects into superorganism hierarchies using either predetermined design strategies or evolved assembly. We envision that these printing methods can provide opportunities for next‐generation autonomous biomanufacturing approaches, enabling the retention, evaluation, and positioning of organisms for a variety of organism‐based applications.

## 4 Experimental Section

### Printing Conditions and Solutions

Details of the printing parameters and nozzles are provided in Table S4 (Supporting Information). Details of solutions (freshwater, seawater, Pluronic hydrogel, and cryoprotectant agents) that were utilized throughout the study are given in Table S5 (Supporting Information).

### Organisms for the Development of Printing Strategies

Five organismal testbeds were used to develop printing strategies. The University of Minnesota Zebrafish Core Facility provided wild‐type zebrafish embryos. Zebrafish embryos were incubated in freshwater at 28 °C and managed with a protocol (# 1506–32642A) documented by the Zebrafish Core‐IACUC. Dinoflagellates (Bio‐Orb, PyroFarms) were incubated in a glass sphere filled with seawater at room temperature. Seawater containing nutrients (DinoNutrients, PyroFarms) was provided every 1.5 weeks. Dinoflagellates were exposed to lighting during the day cycle and left in the dark during the night cycle. Decapsulated brine shrimp embryos (Artemia franciscana, Sustainable Aquatics) were stored in a brine solution at 4 °C before use. Shrimp larvae were obtained by hatching their embryos (Premium Grade Brine Shrimp Eggs, Brine Shrimp Direct) in a hatchery dish (Dohse Aquaristik GmbH & Co. KG) filled with seawater. The dish was placed under lighting for 1 d at room temperature. Black buffalo beetles (D&C Farms) were kept in a critter keeper (Otsumami Tokyo) at room temperature and provided with applesauce (GoGo SqueeZ, Materne).

### Aspiration‐Assisted Adaptive Printing Systems for Organisms

Aspiration‐assisted adaptive printing systems were developed by integrating a multi‐nozzle printing system with machine vision and laser systems. A multi‐nozzle printing system consisted of nozzles connected with pneumatic dispensers (Ultimus V, Nordson EFD), a three‐axis stage (AGS1000, Aerotech) with four independent *z*‐axis heads (MTS25/M‐Z8, Thorlabs), and a desktop computer. A machine vision system was used to detect the 2D locations of organisms and target surfaces. It consisted of lenses (CA‐LH25 and CA‐LH8, Keyence), a vision camera (CA‐200 M, Keyence), a controller (CV‐X322F, Keyence), a power supply (CA‐UA, Keyence), and a lighting system (PLV‐S192, RaLeno). A lens (CA‐LH25) with a minimum working distance of 200 mm was used for zebrafish embryos, dinoflagellates, shrimp embryos, and shrimp larvae. A lens (CA‐LH8) with a minimum working distance of 100 mm was used for the beetles. A laser system was used to measure the profiles of the target surfaces. It consisted of a laser sensor (LK‐H082, Keyence), a controller (LK‐HD500, Keyence), and a power supply (MS2‐H50, Keyence). The machine vision and laser systems were communicated with the printing system via RS‐232 connections. Adaptive printing was programmed in the stage software (A3200, Aerotech).

### Printing of Zebrafish Embryos

The pneumatic dispenser activated vacuum pressure for the picking process (Figure 1B). Pickup times were measured by analyzing slow‐motion videos of the picking process as recorded by a camera (240 FPS, Galaxy Note 8, Samsung).

For the placing process (Figure 1F), a nozzle carrying a zebrafish embryo approached a PP film (61003, C‐Line) on a rigid surface at a speed of 2 mm^ ^s^−1^. The approaching process was recorded with a camera (Nikon D610) and analyzed to determine the critical contact and burst distances.

Axisymmetric FE models (Figure 1D,H) were developed to investigate the stress distributions of the chorion of a zebrafish embryo during picking and placing. Detailed information about the models is presented in Figure S3 (Supporting Information).

The survival rates of zebrafish embryos after printing were assessed (Figure 1E,I). The embryos in the high cell stage (ca. 3 h after fertilization) were prepared. The embryos were incubated at 28 °C after undergoing either the picking process or the pick‐and‐place process. Their survival rates were assessed using the criteria (Table S1, Supporting Information) adopted from previous studies.^[^ ^2^, ^47^ ^]^

The ability of the adaptive printing system to place zebrafish embryos on target locations was tested using circular, square, and hexagonal markers printed on a PET film (ST505, Tekra) by a laser printer (MFC‐L2700DW, Brother) (Figure 1K). The target circular marks had diameters of ca. 2.3 mm.

### Printing of Dinoflagellate‐Laden Droplets

Mesh‐filtered nozzles were made by covering the ends of nozzles with mesh filters. The ends of mesh filters were attached to the sides of nozzles using polyimide electrical tape and epoxy adhesive (14 250, Devcon). A self‐sealing film (PM‐996, Bemis) was then used to cover the attachment site for protection. Detailed information about the mesh filters and nozzles is presented in Table S4 (Supporting Information). The pneumatic dispenser applied vacuum pressure to a mesh‐filtered nozzle to pick up dinoflagellates in a reservoir and applied positive pressure to the nozzle to place the droplets on target surfaces (Figure 2B). The second dispenser applied positive pressure to a stirring nozzle to distribute dinoflagellates in a reservoir before picking. The third pneumatic dispenser applied vacuum pressure to an auxiliary nozzle to aspirate unwanted drops of a residual solution from the side of a mesh‐filtered nozzle during the stage movement. The side view images of dinoflagellate‐laden droplets placed on PP films were captured by a camera (Nikon D610) to calculate their volumes using a spherical cap formulation. The number of dinoflagellates in the droplets was counted as described in Figure S8 (Supporting Information).

After the pick‐and‐place process, the survival rates of the dinoflagellates were investigated (Figure 2C). The placed dinoflagellates were treated with a 0.4% trypan blue solution (T8154‐20ML, Sigma) for 5 min and then rinsed with seawater. The blue staining of dinoflagellates under a microscope (Leica DM4000) indicated their non‐viability (Figure S9, Supporting Information).

### Printing of Shrimp Embryo‐ and Larva–Laden Droplets

The pneumatic dispensers were programmed to apply either vacuum pressure or positive pressure to mesh‐filtered, auxiliary, and stirring nozzles (Figure 2D,F) to create dinoflagellate‐laden droplets. PEG solutions were prepared by mixing seawater with PEG (Alfa Aesar). Shrimp embryo‐ and larva–laden droplets (seawater, 10% w/w PEG, and 20% w/w PEG) were placed on PP films. Their droplet volumes were measured using the same method as for dinoflagellate‐laden droplets. The numbers of embryos and larvae in their droplets were measured as illustrated in Figure S8 (Supporting Information).

Survival rates of shrimp embryos and larvae were evaluated after picking and placing. Their seawater, 10% w/w PEG, and 20% w/w PEG droplets were placed on PP films (Figure 2E,G). The placed embryos and larvae were washed and incubated in seawater at 28 °C. The embryos were considered alive if they hatched and could swim 1 d after incubation. The larvae were considered alive if they could swim 1 d after incubation.

### Printing of Beetles

Beetles were immersed in 70% ethanol (861 261, Carolina Biological Supply Company) for more than 5 min to immobilize them. The pneumatic dispenser was used to apply different vacuum pressures to a nozzle to pick up the beetles (Figure 3B). The pickup times of the beetles by the nozzle were evaluated using slow‐motion videos of the picking process as captured by a camera (240 FPS, Galaxy Note 8, Samsung).

The tracking errors of the machine vision‐guided adaptive printing system were evaluated with a moving circular target (diameter: ca. 16 mm) (Figure 3C). The circular target was moved by a motorized stage (MTS25/M‐Z8, Thorlabs) at different speeds while a nozzle tracked it (Figure S14, Supporting Information). The nozzle printed one dot on the circular target before it began to move and the other dot while it was in motion, using a mixture of 40% w/v Pluronic hydrogel and a food coloring agent (black color, Ktdorns). The distance between the dots was used to measure the tracking errors.

40% w/v Pluronic hydrogel structures with different thicknesses were printed to immobilize beetles at target locations (Figure 3D). The displacement of a beetle placed in a hydrogel structure using a nozzle was calculated by tracking its position with the machine vision system, as shown in Figure S21A (Supporting Information).

Survival rates of beetles were investigated after the pick‐and‐place process and removal of the Pluronic hydrogel used to fix them (Figure 3E). The experiments involved two groups of beetles: one group was picked and placed in a petri dish, and the other group was picked and placed in 40% w/v Pluronic hydrogel, after which they were removed from the hydrogel. Both groups were incubated at room temperature for 1 d without any feeding. The beetles that exhibited movement were considered to have survived.

### Cryopreservation and Laser Warming of Zebrafish Embryos

The multi‐nozzle adaptive printing system picked up zebrafish embryos in freshwater, placed them on cryotop devices, and dispensed cryoprotectant droplets (ca. 3 µL) on them (Figure 4A‐i). The cryotop device was fabricated by creating a flat tip with a PP film and connecting the tip to a wooden rod. Three cryotop devices with different heights (ca. 4.6 mm, ca. 7.9 mm, and ca. 11.1 mm) were randomly placed. The components of the cryoprotectant agent were identical to those of cryoprotectant agent B introduced in Table S5 (Supporting Information), but the GNRs were not included. The cryoprotectant agent was mixed with food coloring agents (green and red colors, Ktdorns) for enhanced visualization.

The cryopreservation and laser rewarming methods used for zebrafish embryos placed by the adaptive printing system were adapted from previous studies that involved manual manipulation of the embryos^[^ ^1^, ^2^ ^]^ (Figure 4A‐ii). At the University of Minnesota Zebrafish Core Facility, 9 and 72 nL of GNR‐included cryoprotectant agent A were delivered into the yolk and perivitelline fluid of zebrafish embryos in the high cell stage via microinjection, respectively. The adaptive printing system placed the microinjected embryos on cryotop devices and dispensed GNR‐included cryoprotectant agent B droplets (ca. 3 µL) on them. The embryos were in the droplets for 300 s and vitrified by plunging them into liquid nitrogen for 120 s. The embryos were then removed from liquid nitrogen, positioned at the focal point of a laser (i980w, LaserStar, Inc.), and rewarmed via a single laser pulse (power: 300 V and pulse time: 1 ms). The rewarmed embryos were immersed in a post‐warming bath for 600 s, rinsed 3 times in freshwater, and incubated in petri dishes filled with freshwater at 28 °C. The survival rates of the rewarmed embryos were evaluated based on the criteria and observation times used in the previous studies (Table S1, Supporting Information).^[^ ^2^, ^47^ ^]^ The components of the cryoprotectant agents A and B and the post‐warming bath are detailed in Table S5 (Supporting Information).

### Cryopreservation and Convective Warming of Shrimp Embryos

The adaptive printing system with a mesh‐filtered nozzle placed shrimp embryo–laden cryoprotectant droplets on cryotop devices (Figure 4B‐i). The components of the cryoprotectant agent can be found in Table S5 (Supporting Information). Three cryotop devices were randomly positioned at different heights (ca. 4.6 mm, ca. 7.9 mm, and ca. 11.1 mm).

Shrimp embryos placed by the adaptive printing system were cryopreserved and rewarmed (Figure 4B‐ii). Shrimp embryos were placed in a reservoir filled with the cryoprotectant agent for 600 s before printing. Embryo–laden cryoprotectant droplets placed on cryotop devices were submerged in liquid nitrogen for 60 s for vitrification. Following removal from liquid nitrogen, the embryos were convectively rewarmed in seawater at room temperature. They were rinsed 3 times and incubated in seawater at 28 °C. Their survival rates were assessed based on hatching and their swimming ability after 1 d.

### Sorting and Placing Live Zebrafish Embryos on Curved Surfaces

Live and dead zebrafish embryos and fluorescent microspheres were prepared in freshwater (Figure 4C‐i). Dead embryos were obtained from the University of Minnesota Zebrafish Core Facility. The red (UVPMS‐BR‐0.995, Cospheric) and yellow (UVPMS‐BY2‐1.00, Cospheric) microspheres had diameters ranging from 710 to 850 µm and from 1 to 1.2 mm, respectively, according to the provided seller descriptions. A curved surface was prepared by bending a copper foil sheet (LTKJ LTD) to a curvature of 0.35 (Figure 4C‐ii). The adaptive printing system placed zebrafish embryos on the curved sheet at a spacing interval of 4 mm.

### Dinoflagellate‐Based Spherical and Planar Displays

To fabricate a dinoflagellate‐based spherical display (Figure 4D‐ii), a hemispherical glass dome with a diameter of ca. 15 mm was prepared by cutting a borosilicate glass flask (ML‐1110‐708, Wilmad‐LabGlass). Chambers were conformally printed on the inside surface of the glass dome using clear RTV silicone (LOCTITE SI 595, Henkel). The distance between the central and side chambers was 8 mm. The conformal toolpaths for printing the chambers were generated by dividing a circle into segments of 2° and determining the *z*‐coordinates for each segment using the equation of a sphere. To create a dinoflagellate‐based planar display (Figure 4D‐iv), a 5 × 9 array of silicone chambers was printed on a transparent PET film with a spacing of 7.5 mm. Dinoflagellates in seawater were picked and placed in each chamber two times using the printing conditions presented in Table S4 (Supporting Information).

The spherical and planar displays were connected to a speaker (LEPAZIA59224, Walfront) and an amplifier (F900S, Facmogu) to test their responses to audio‐induced vibrations. During testing, the displays were recorded using a camera (Nikon D750). The videos were then analyzed in MATLAB (MathWorks) to obtain the normalized grayscale intensities of the dinoflagellate‐laden chambers and the normalized amplitude of the soundtrack (Figure S20, Supporting Information). When measuring the normalized intensities, a circular mask was used to crop each chamber (Shotcut, Meltytech), and the background intensity was calibrated to a value of 0.

### Beetles with Integrated Symbols and LEDs

A 40% w/v Pluronic hydrogel was used to fix beetles placed by the adaptive printing system. Invisible UV‐responsive hydrogel was prepared by combining UV inkjet printing ink (UV 365 nm reaction, UVstuff) with 40% w/v Pluronic hydrogel at a concentration of 2% v/v. The hydrogel was used to conformally print secret symbols on beetles (Figure 4E‐ii). The symbols comprised three layers, each with a thickness of 60 µm. They were displayed via illumination by UV light from a flashlight (00F‐51UV‐001A, Escolite).

The top and bottom electrodes were conformally printed on beetles using a silver conductive epoxy adhesive (8330S, MG chemicals) (Figure 4E‐iii). Surface‐mounted red LEDs (0805, Chanzon) were interfaced with the electrodes. LEDs integrated with the beetles were turned on using a Tesla coil (YS03, Joytech).

### Statistical Analysis

The non‐parametric Mann–Whitney U test was employed to compare the survival rates of organisms between experimental and control groups. Organisms that were individually manipulated were grouped for analysis. The survival rates of zebrafish embryos were assessed with 5 groups of 6 samples each (*n* = 30) for Figure 1E and 10 groups of 10 samples each (*n *= 100) for Figure 1I. The survival rates of the beetles in Figure 3E were assessed with 5 groups of 6 samples each (*n* = 30). All statistical analyses were conducted using MATLAB (MathWorks). A significance level of 5% was used for all tests.

## Conflict of Interest

M.C.M., G.H., J.C.B., K.K., and K.T.S. are inventors on a pending U.S. patent application (US17/813779) related to this work filed by the University of Minnesota on 20 July 2022. M.C.M. serves on the Scientific Advisory Board and holds equity in GRIP Molecular Technologies. M.C.M. is Co‐Founder and CSO of Flui3D Inc. These interests have been reviewed and managed by the University of Minnesota in accordance with its Conflict of Interest policies. The authors declare that they have no other competing interests.

## Supporting information

Supporting Information

Supplemental Movie 1

Supplemental Movie 2

Supplemental Movie 3

Supplemental Movie 4

Supplemental Movie 5

Supplemental Movie 6

Supplemental Movie 7

Supplemental Movie 8

Supplemental Movie 9

## Data Availability

All data needed to evaluate the conclusions in the paper are present in the paper and/or the Supporting Information. Additional supporting data are available at the Data Repository for the University of Minnesota (https://doi.org/10.13020/qkm1‐4913) and from the authors upon request.
