# Supplementary material for: 3D Printed Organisms Enabled by Aspiration‐Assisted Adaptive Strategies
Source: Adv Sci (Weinh). 2024 Jun 21;11(32):2404617. doi: 10.1002/advs.202404617 (PMC11348114; doi:10.1002/advs.202404617)
Supplement: Supplementary file 1 — Supporting Information [file ADVS-11-2404617-s008.docx]

Supporting Information

3D Printed Organisms Enabled by Aspiration-Assisted Adaptive Strategies

Guebum Han, Kanav Khosla, Kieran T. Smith, Daniel Wai Hou Ng, JiYong Lee, Xia Ouyang, John C. Bischof, Michael C. McAlpine*

Guebum Han, Kanav Khosla, Daniel Wai Hou Ng, JiYong Lee, Xia Ouyang, John C. Bischof, Michael C. McAlpine

Department of Mechanical Engineering, University of Minnesota, Minneapolis, MN 55455, USA.

E-mail: mcalpine@umn.edu

Guebum Han, Kanav Khosla, Kieran T. Smith, John C. Bischof, Michael C. McAlpine

Center for Advanced Technologies for the Preservation of Biological Systems (ATP-Bio), University of Minnesota, Minneapolis, MN 55455, USA.

Kieran T. Smith

Department of Fisheries, Wildlife and Conservation Biology, University of Minnesota, Minneapolis, MN 55108, USA.

John C. Bischof, Michael C. McAlpine

Department of Biomedical Engineering, University of Minnesota, Minneapolis, MN 55455, USA.

**Supporting Note S1.** Measurement of elastic moduli of shrimp embryos and shrimp larvae.

Equilibrium moduli of shrimp embryos and larvae were measured by performing confined compression tests. Shrimp embryos and larvae were prepared in the same manner as described in the Methods section, and then filtered from seawater using a nylon filter with a mesh size of 60 μm (ME 17228, Tisch Scientific). The filtered samples were transferred into a confined chamber. Equilibrium force responses (MS304S, Mettler Toledo) of shrimp embryos and larvae were measured at strains of 3% and 6% under confined compression with a flat-ended cylindrical indenter (radius: 2.35 mm) after a relaxation time of 100 s. During the relaxation time, seawater (liquid phase) flowed freely through the permeable boundary of the indenter, such that fluid pressure did not affect the equilibrium force responses. This was confirmed by observing that the equilibrium force responses were nearly zero in the absence of shrimp embryos and larvae. Shrimp embryos and larvae remained inside the chamber and were deformed by the indenter. As a result, the equilibrium force responses were dominantly from the shrimp embryos and larvae. The equilibrium stress responses were calculated by dividing the equilibrium force responses by the cross-sectional area of the indenter. The relaxation time of 100 s was sufficiently long to ensure that the stress responses reached equilibrium states (stress change between 100 s and 105 s: 0.55 ± 0.13% for shrimp embryos and 1.53 ± 0.71% for shrimp larvae). Linear functions were fitted to equilibrium stress-strain curves from 0% to 6% (R^2^ $\geq$0.96,

Figure S1A). The slopes represented aggregate moduli of shrimp embryos and larvae. The aggregate moduli, *H_A_*, were converted to elastic moduli, *E*, by ^[1]^:

1. $E=\frac{(1+v)(1-2v)}{1-v}H_{A}$

where *v* is Poisson’s ratio. Poisson’s ratios of shrimp embryos and larvae were assumed to be 0.3. Using this equation, the elastic moduli of shrimp embryos and larvae were determined to be 164.61 ± 23.56 kPa and 16.66 ± 1.16 kPa (*n* = 3), respectively.

**Supporting Note S2.** Finite element (FE) model of chorion of zebrafish embryo.

Axisymmetric FE models of the chorion and nozzle were developed to investigate the stress distributions of the chorion during the pick-and-place printing process. For the picking process (Figure S3A), the chorion (diameter: 1.20 ± 0.11 mm, *n* = 5), was modeled based on its optical microscope images. The thickness of the chorion was modeled to be 3 μm ^[2]^. The nozzle was modeled with an inner diameter of 510 μm with a fillet radius of 10 μm and an outer diameter of 780 μm with a fillet radius of 65 μm based on optical microscope observations. The chorion was modeled to be a flexible (268 linear quadrilateral elements) linear elastic solid (elastic modulus = 1.51 MPa and Poisson’s ratio = 0.5 ^[2]^). The printer nozzle was modeled to be rigid. The contact condition between the chorion and the nozzle was set to be frictionless. A fluid cavity interaction was employed to maintain a constant volume of the chorion, while a vacuum boundary condition was applied to the chorion inside the nozzle. The FE-predicted stress distribution of the chorion was examined at vacuum levels of 2.5 kPa and 4 kPa.

For the placing process (Figure S3B), a rigid substrate was added to the FE models used for the picking process. The nozzle was subjected to a displacement boundary condition to compress the chorion. A vacuum boundary condition was not applied to match the experimental condition. The contact condition between the chorion and the substrate was frictionless. The FE-predicted stress distribution of the chorion was obtained under compression by the nozzle. All FE simulations were conducted in Abaqus FEA.

**Supporting Note S3.** Image processing filters for machine vision.

The incorporation of a machine vision system allowed a three-dimensional (3D) printing system to adjust to updated information regarding target organisms and substrates. In a machine vision system, image processing filters were used to highlight the targets in the raw images to ensure their accurate detection. The filter parameters were fine-tuned through observation, and these parameters were updated according to the printing environment, such as the indoor lighting level. A machine vision-enabled adaptive printing system detected the target organisms and substrates via a pattern search function on the filtered images. A summary of the filters and search function can be found in Table S2.

**Supporting Note S4.** Time required to prepare organism-included cryoprotectant droplets for cryopreservation.

Zebrafish embryos: An adaptive printing system created a zebrafish embryo-included cryoprotectant droplet for vitrification in ca. 13 s. The process comprised two steps: ca. 8 s for placing an embryo on a cryotop device and ca. 5 s for dispensing a gold nanorod (GNR)-included cryoprotectant droplet on the placed embryo. The first step involved: (1) the acquisition of the locations of the embryo and cryotop device using the machine vision and laser systems, (2) the pick-and-place printing process, (3) removal of residual water in the nozzle, and (4) the stage movement. The second step involved: (1) cryoprotectant droplet printing on the embryo and (2) the stage movement.

The manual preparation time for a zebrafish embryo-included cryoprotectant droplet for vitrification was ca. 150 s ^[3,4]^. The process consisted of three steps: ca. 60 s for collecting and positioning the embryo on the cryotop device using a pipette, ca. 60 s for removing excess water using tissue paper, and ca. 30 s for dispensing the cryoprotectant with a pipette. During the first step, a zebrafish embryo was sucked into a pipette with freshwater and placed on a cryotop tip with excess freshwater. The embryo should be positioned at the center of the cryotop tip to ensure precise laser focusing during the rewarming process.

Shrimp embryos: An adaptive printing system with a mesh-filtered nozzle generated a shrimp embryo-laden cryoprotectant droplet for vitrification in ca. 20 s. The process involved: (1) the acquisition of the locations of the reservoir and cryotop devices with the machine vision and laser systems, (2) the pick-and-place printing process, (3) removal of residual solution in the mesh-filtered nozzle, and (4) the stage movement.

The manual protocol to prepare a shrimp embryo-laden cryoprotectant droplet required ca. 150 s. The protocol comprised three steps: ca. 60 s for collecting and placing shrimp embryos on a cryotop device with a pipette, ca. 60 s for removing seawater around embryos with tissue paper and gathering scattered embryos into the center of the cryotop tip, and ca. 30 s for dispensing a cryoprotectant droplet on the embryos with a pipette.


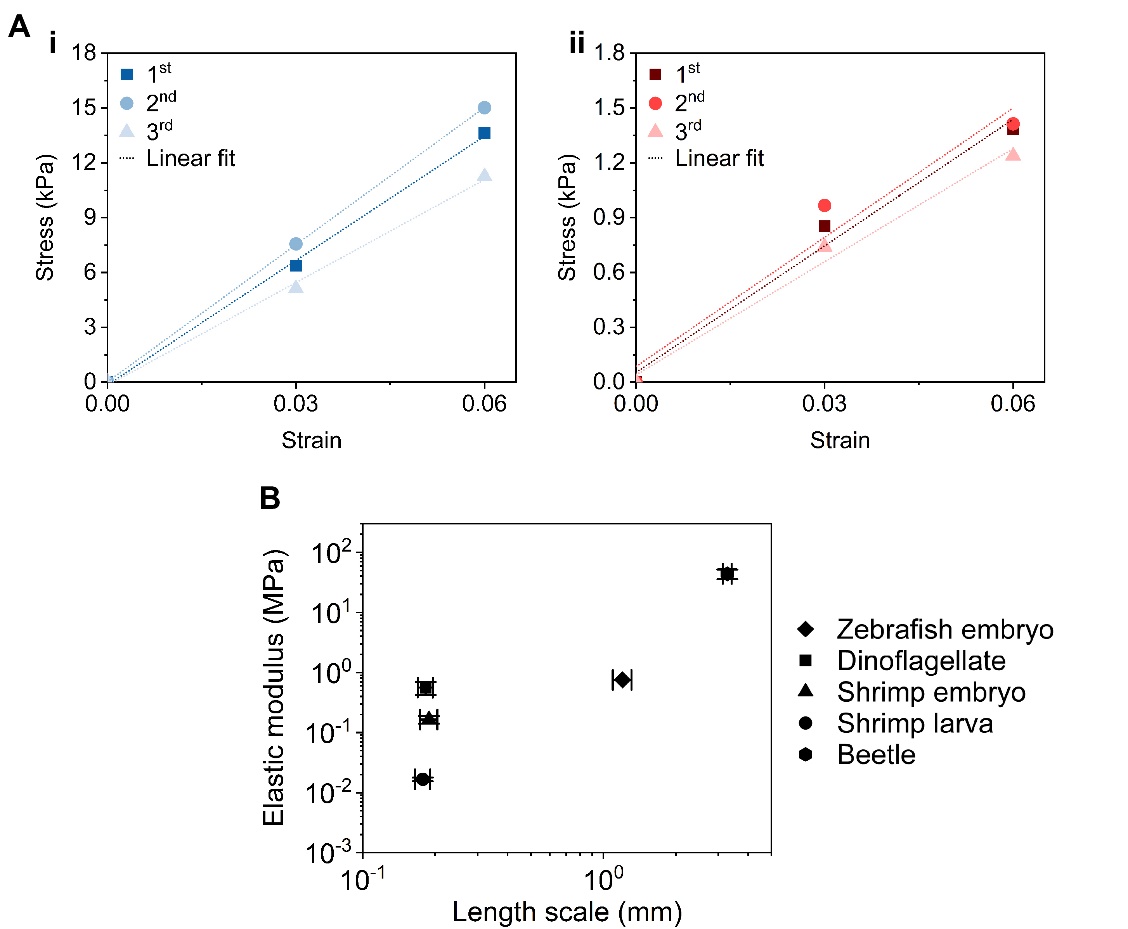


Figure S1. Mechanical properties of organisms. A) Measurement of equilibrium elastic moduli of shrimp (i) embryo and (ii) larvae (linear fits: R^2^ $\boldsymbol{\geq}$0.96) (*n* = 3). More detailed information about the measurement is available in Supporting Note S1. B) Elastic moduli and length scales of organisms used for the development of aspiration-assisted printing strategies. Elastic moduli of zebrafish embryos ^[2,5]^, dinoflagellates ^[6]^, and beetles ^[7]^ were obtained from previous studies. Only the average value for zebrafish embryos was reported since the standard deviation was unavailable. The length scales were determined by measuring the shortest body lengths of organisms (*n* = 5). Since a zebrafish embryo is spherical, the length scale corresponds with its diameter.


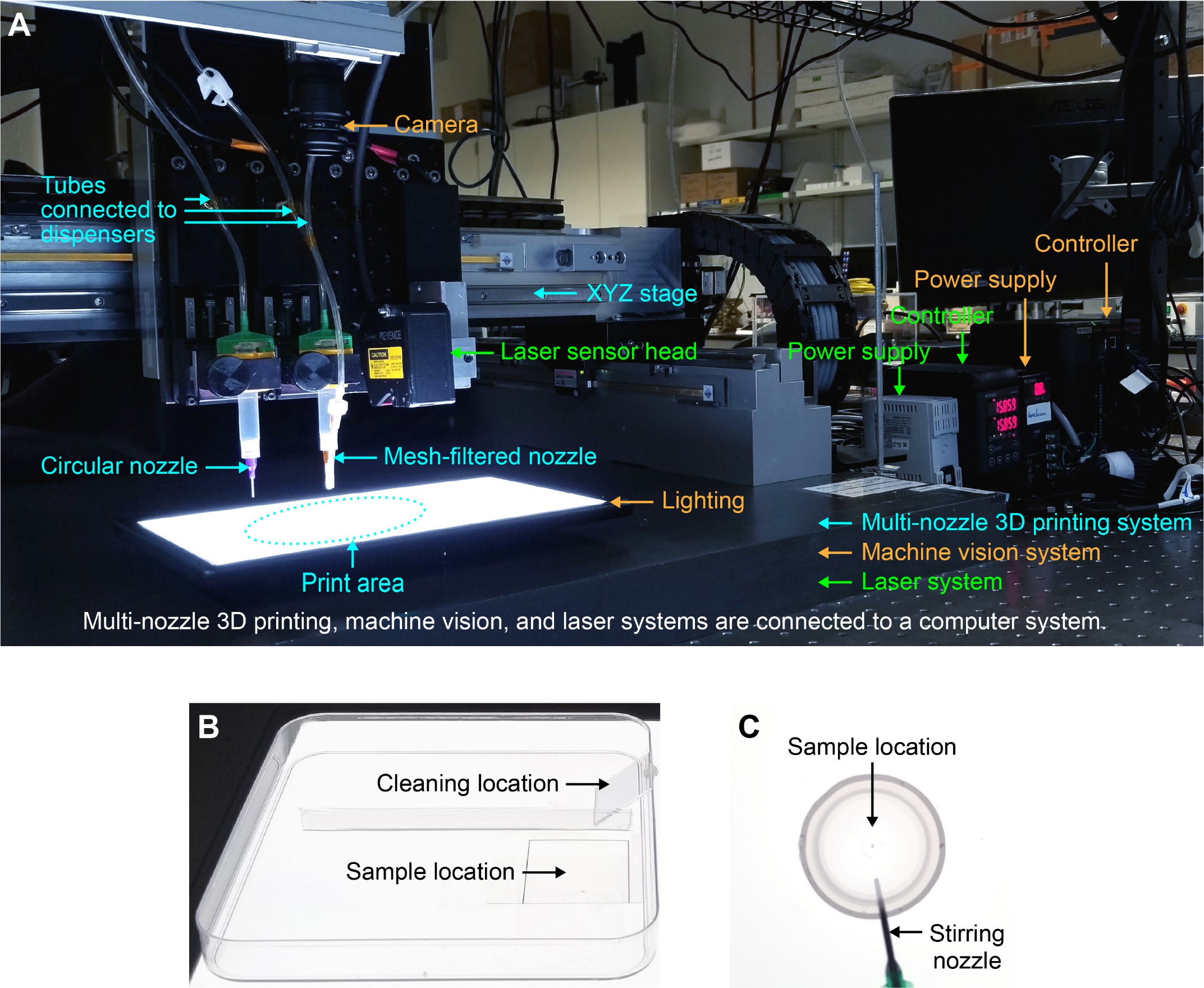


Figure S2. Aspiration-assisted adaptive printing system developed for zebrafish embryos, [dinoflagellates,](https://en.wikipedia.org/wiki/Dinoflagellate) shrimp embryos, and shrimp larvae. A) Image of adaptive printing system. The adaptive printing system, which was guided by machine vision and laser systems, continuously responded to updated spatial and visual information about organisms and target substrates. B) Image of reservoir for zebrafish embryos. C) Image of reservoir for dinoflagellates, shrimp embryos, and shrimp larvae. The stirring nozzle was programmed to apply compressed air to the reservoir to distribute organisms.


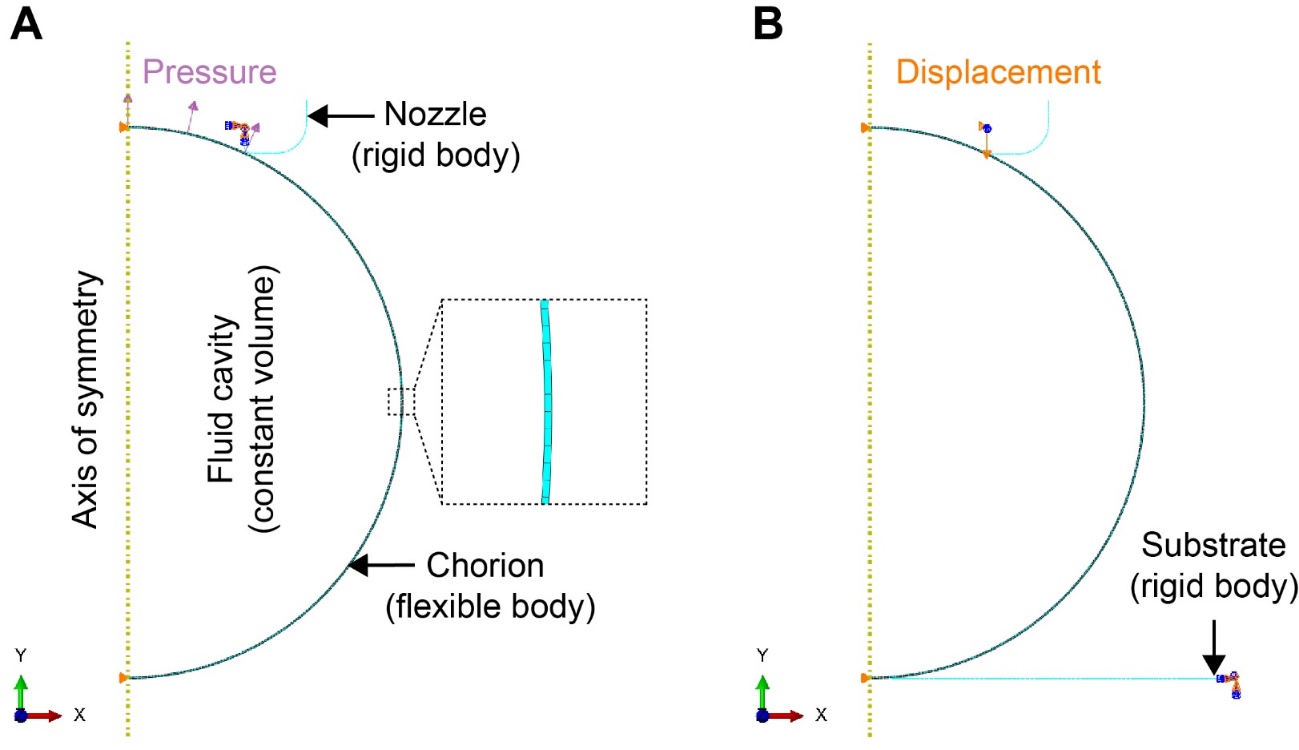


Figure S3. FE modeling for the chorion of the zebrafish embryo, the nozzle, and the substrate. A) Picking and B) placing processes of an embryo. Details of FE models are given in Supporting Note S2.


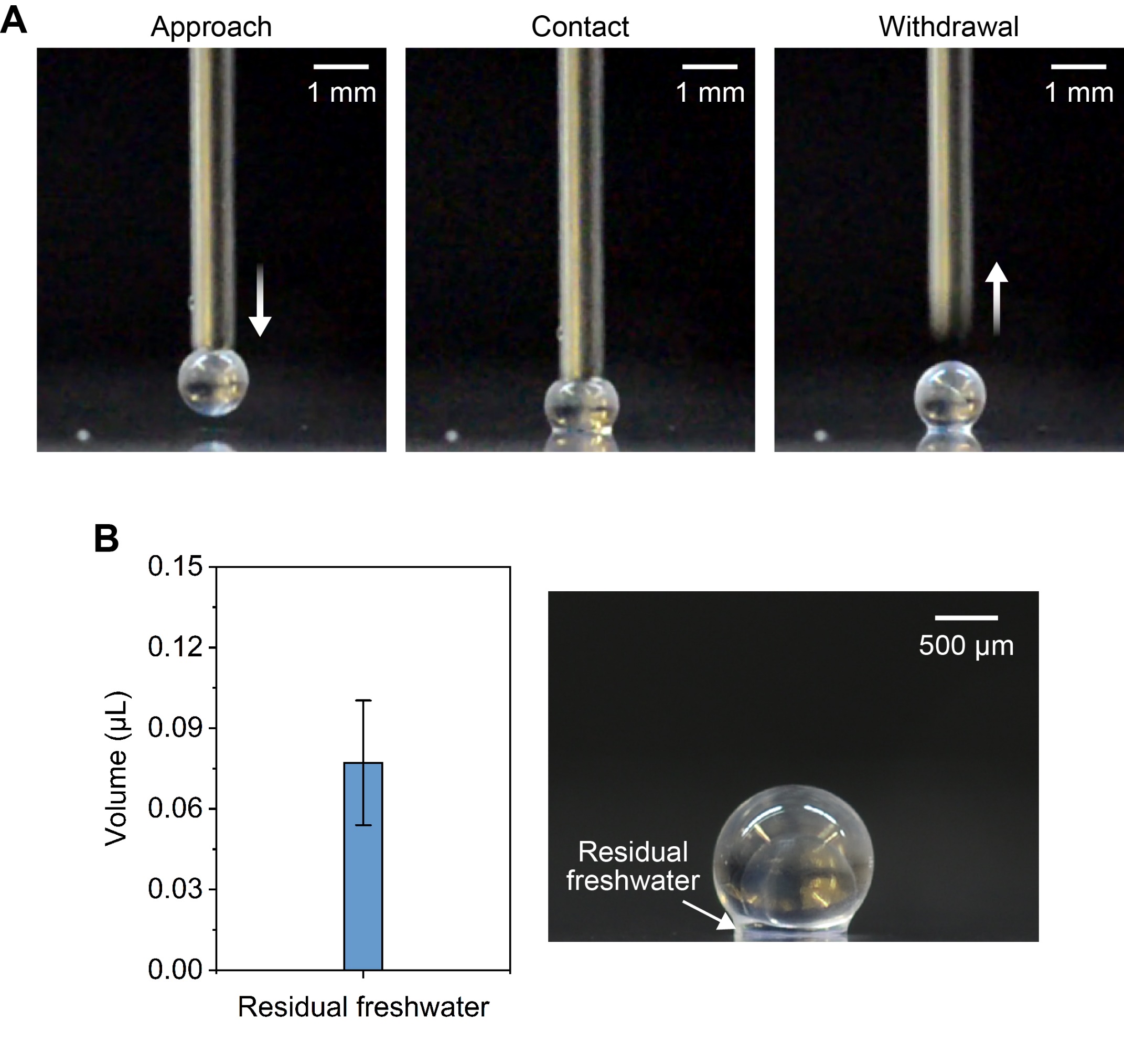


Figure S4. Investigation of the placing process for zebrafish embryos. A) Images of placing zebrafish embryos at different instances during the process. The distance between the end of the nozzle and substrate at the contact moment was 800 μm. B) Volume of residual freshwater around placed embryos (*n* = 10). The volume of the residual freshwater was determined by estimating the nominal volume of residual freshwater with a cylindrical volume formula, estimating the volume of an embryo immersed in the residual freshwater with a spherical cap formula, and subtracting the volume of the immersed embryo from the nominal volume of the residual freshwater. The image analysis was performed in ImageJ.


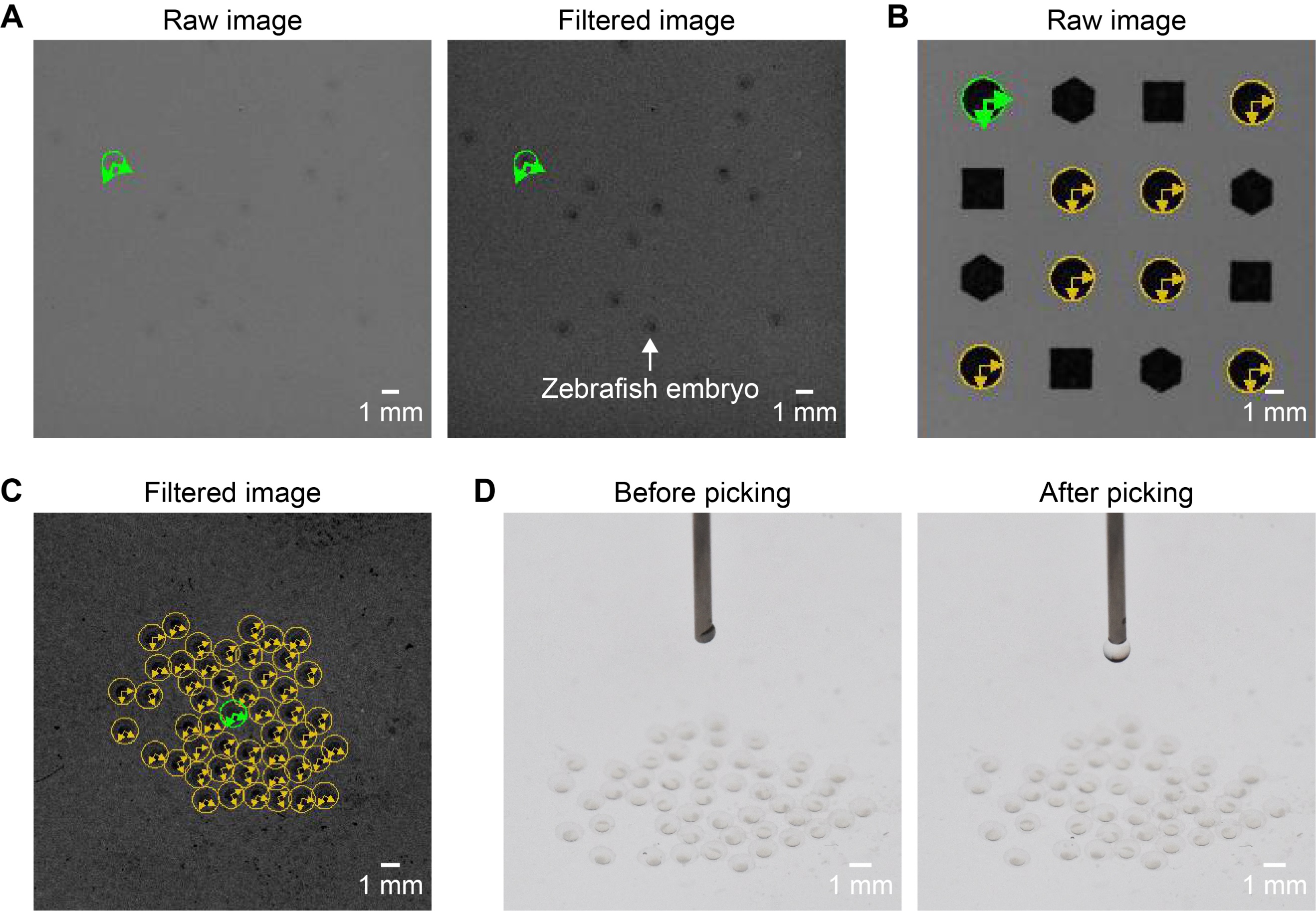


Figure S5. Machine vision system used for placing zebrafish embryos. A) Raw and filtered images of zebrafish embryos in freshwater from the machine vision system. The image captured by a camera was processed via a contrast conversion filter to enhance the detection of zebrafish embryos (filtering parameter values: offset = - 87 and span = 3). The filtered image was used to detect zebrafish embryos via a pattern search function based on a reference image (detection conditions: search sensitivity = 100%, accuracy = 100%, and minimum match percentage = 10%). A green circle highlighted one of the detected zebrafish embryos. B) Raw image of circular, hexagonal, and square marks printed on polyethylene terephthalate (PET) film from the machine vision system. The raw image captured by a camera was used to find eight circular target marks via a pattern search function to selectively place embryos on the targets (detection conditions: search sensitivity = 100%, accuracy = 100%, and minimum match percentage = 10%). Green and yellow circles represent circular target markers detected by the machine vision system. The green circle indicates the first mark location transferred to the printing system. C) Filtered image showing the vision detection of zebrafish embryos at a high concentration. The filtering parameters and pattern search function were identical to those of A). Green and yellow circles represent embryos detected by the machine vision system. The green circle shows the first embryo location transferred to the printing system. D) Images showing the picking of an embryo from a highly concentrated population. The printer nozzle, which was smaller than the embryo, picked up the embryo from a highly concentrated population. After the target embryo was removed, the surrounding embryos moved slightly within the reservoir. The vision system updated the altered locations at each cycle. Details of the filter and function used in the machine vision system are provided in Supporting Note S3 and Table S2.


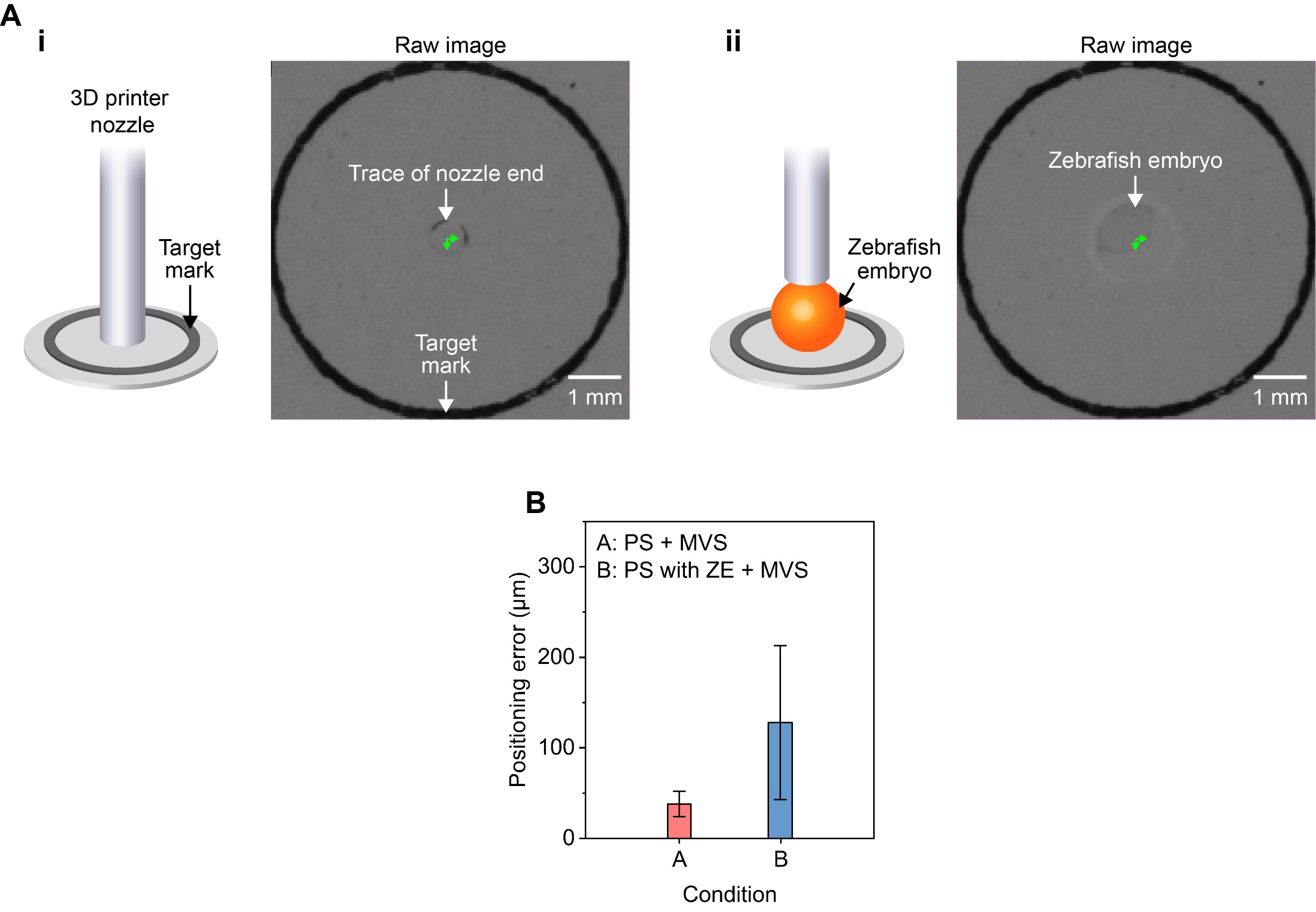


Figure S6. Estimation of the positioning error of the machine vision-guided adaptive printing system for zebrafish embryos. A) Illustrations and raw images of experimental setups (i) without and (ii) with zebrafish embryos. (i) Using the machine vision-guided adaptive printing system, a circular nozzle end was dipped in a food coloring agent (black color, Ktdorns) and was positioned on a circular target mark printed on a PET film. This resulted in the nozzle leaving a trace on the PET film. (ii) Zebrafish embryos were picked and placed on the circular target mark by the machine vision-guided adaptive printing system. The pattern search function of the machine vision system (detection conditions: search sensitivity = 100%, accuracy = 100%, and minimum match percentage = 10%) (Table S2) was used to detect the circular target mark. B) Positioning errors of zebrafish embryos (PS: printing system, MVS: machine vision system, and ZE: zebrafish embryo) (*n* = 10). To determine the centers of the nozzle trace, zebrafish embryo, and circular mark, circles were fitted to them using ImageJ. The circles were uniquely defined by selecting three points on each of them. Positioning errors were calculated by measuring the difference between the centers of the nozzle trace and embryo and the centers of the target marks. The measurement resolution was ca. 14 µm, based on the size of a single pixel.


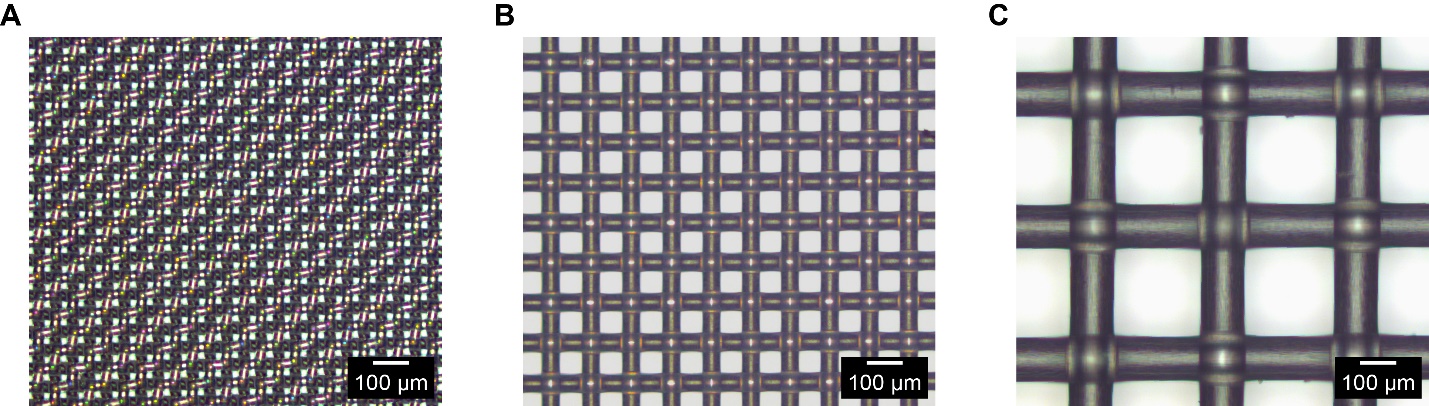


Figure S7. Optical microscope images of the mesh filters for organisms. Sizes of nylon mesh filters: A) 18 μm, B) 60 μm, and C) 250 μm. The filter performance for organisms is shown in Table S3.


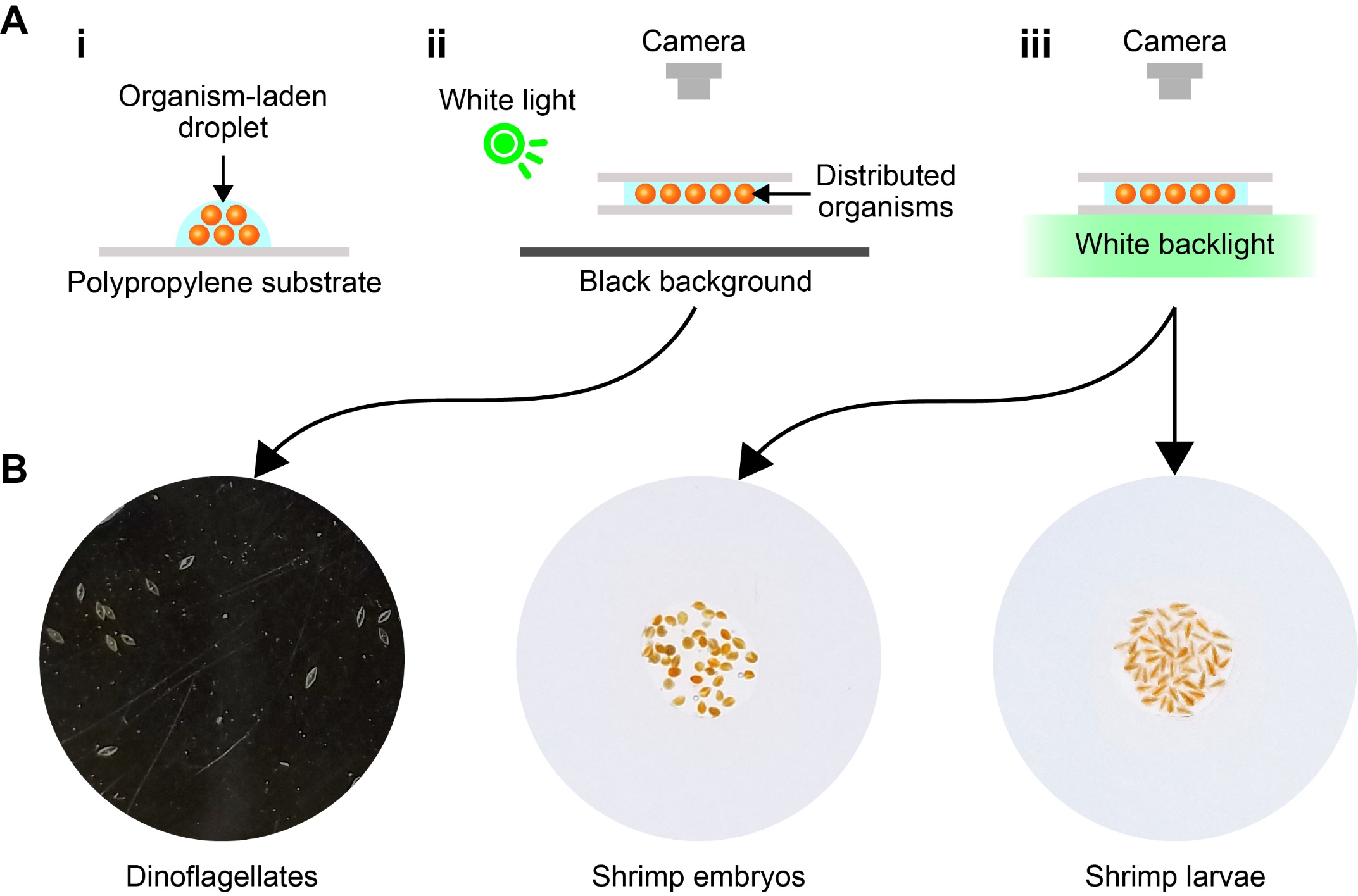


Figure S8. Counting multiple organisms placed in droplets. A) (i) Organism-laden droplets were placed on a polypropylene (PP) film. These droplets were spread out between the PP films. The organisms were then observed against either a (ii) black or (iii) white background, depending on their transparency. B) Representative images of dinoflagellates, shrimp embryos, and shrimp larvae used for counting.


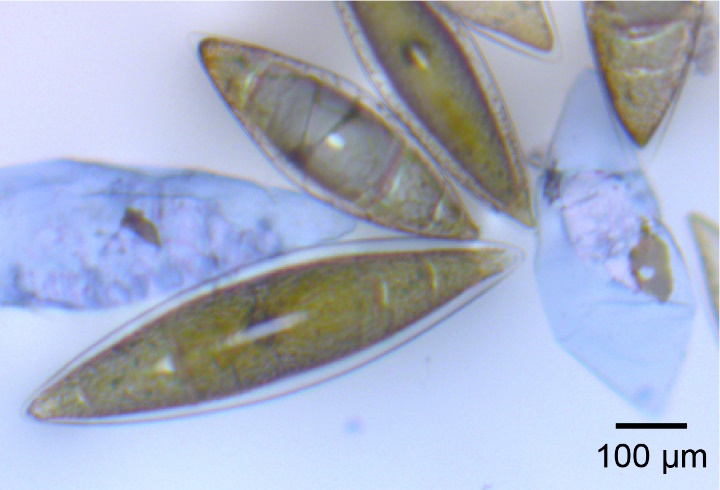


Figure S9. Image of dinoflagellates after trypan blue staining assay. The assay stained dead dinoflagellates.


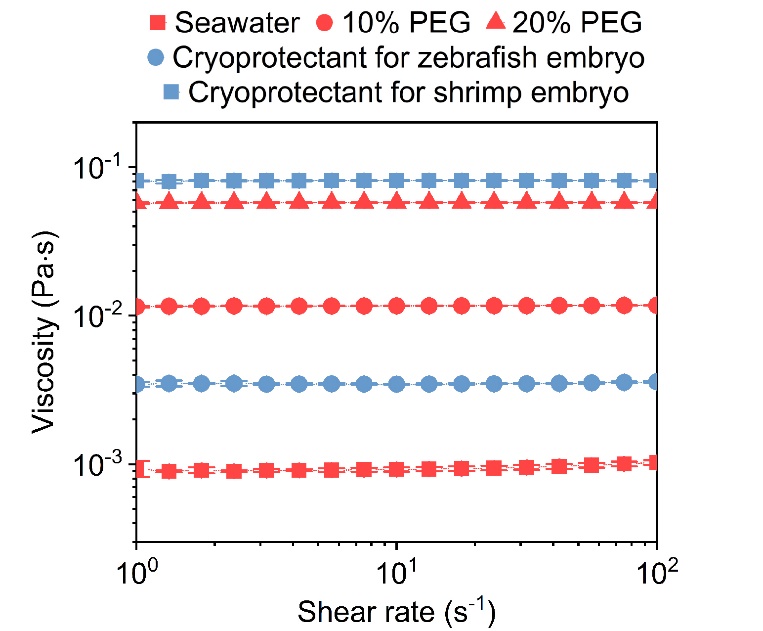


Figure S10. Viscosities of the solutions used in the printing process. The solutions were evaluated using *n* = 3 (PEG: polyethylene glycol).


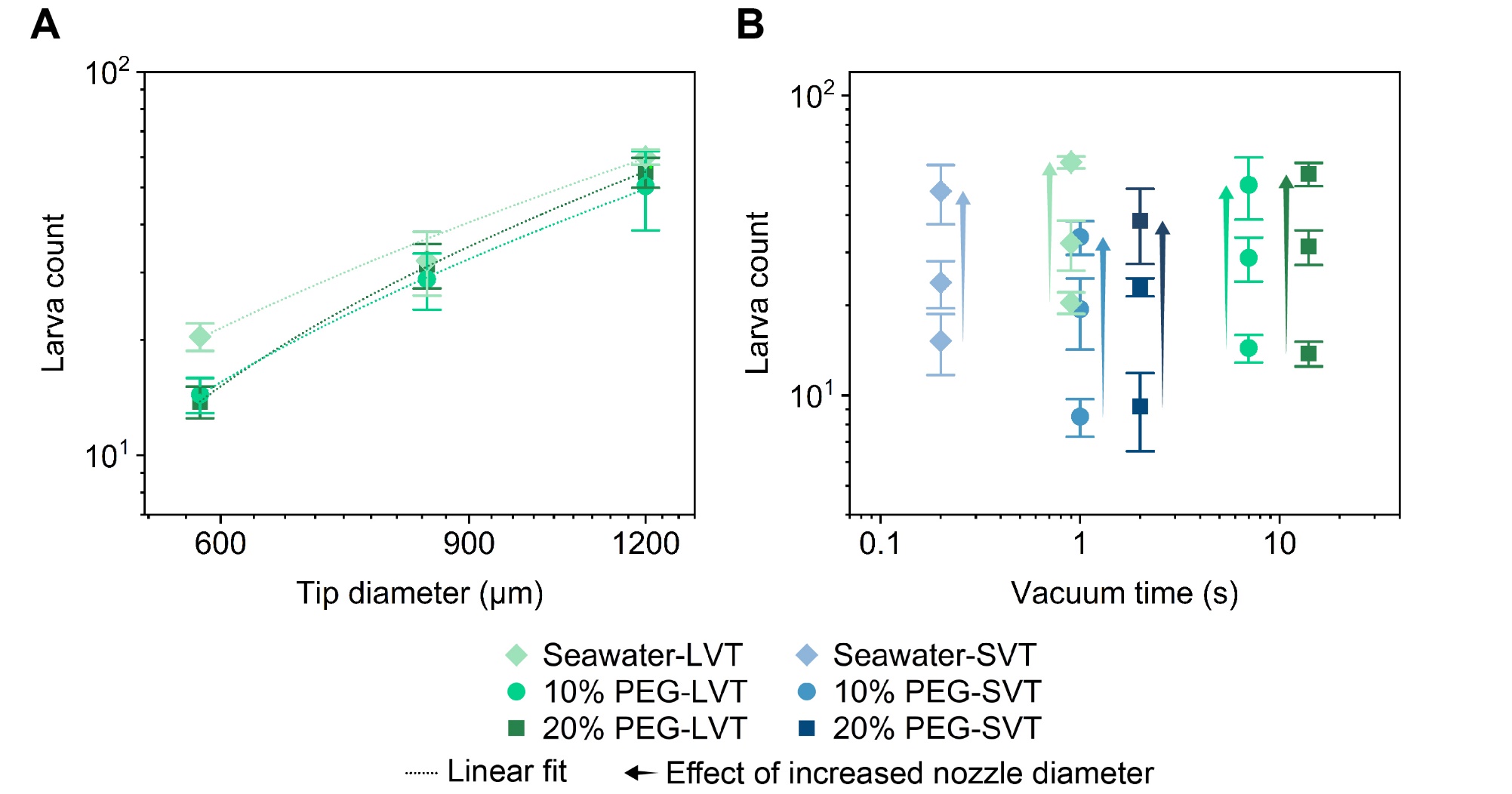


Figure S11. Effect of the vacuum time and the nozzle diameter on the picking of shrimp larvae. A) The number of shrimp larvae picked using mesh-filtered nozzles was measured under long vacuum times (LVTs) (*n* = 5). B) The result was then compared to the number of larvae picked under short vacuum times (SVTs) (*n* = 5). The effects of nozzle diameters on the number of larvae were highlighted with arrows. The mesh-filtered nozzles used for both LVTs and SVTs were identical.


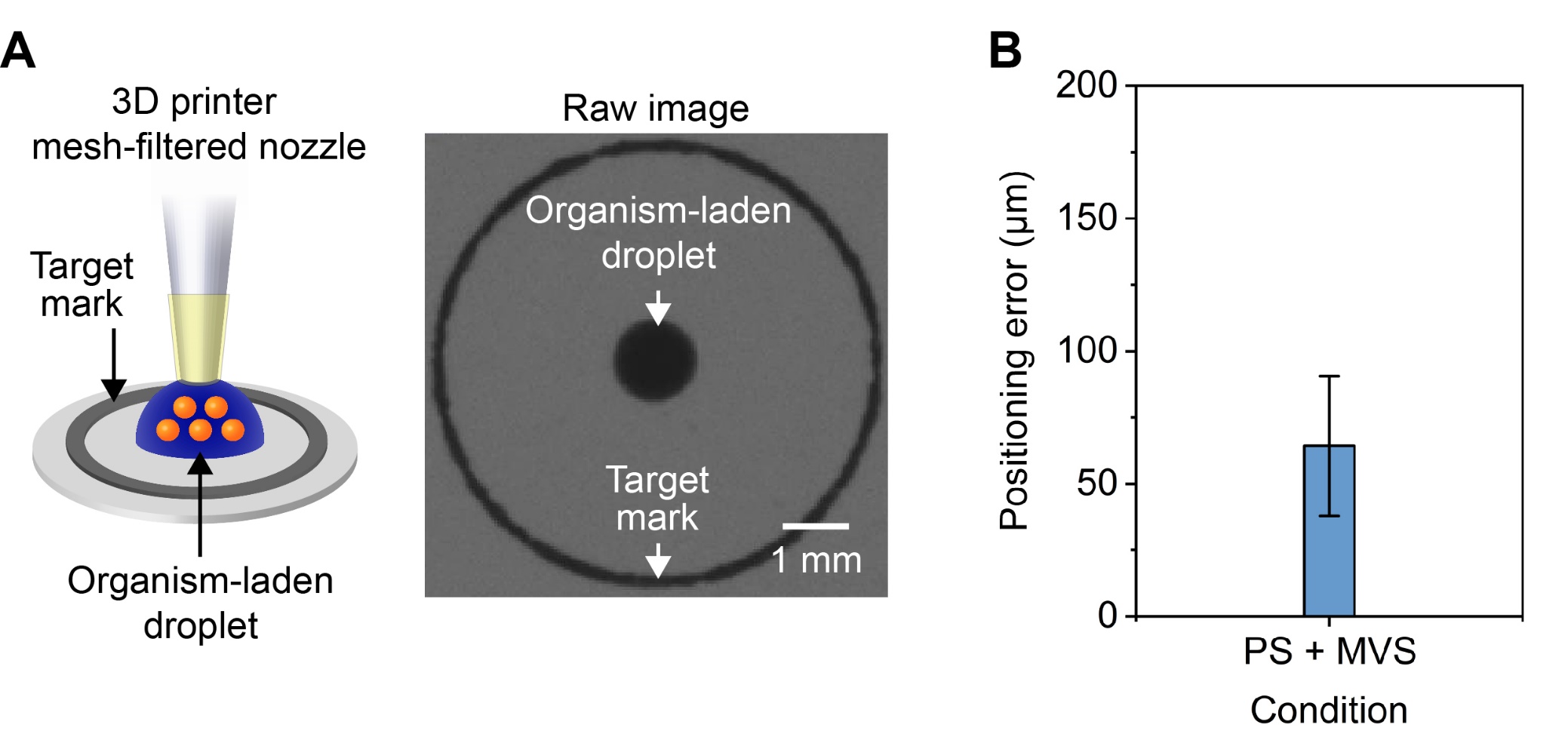


Figure S12. Estimation of the positioning error of the machine vision-guided adaptive printing system for organism-laden droplets. A) Illustration and raw image of experimental setup. Blue food coloring was added in 10% w/w PEG to enhance the visualization of the droplet. The pattern search function of the machine vision system (detection conditions: search sensitivity = 100%, accuracy = 100%, and minimum match percentage = 10%) (Table S2) was used to detect the target mark printed on a PET film. A shrimp embryo-laden 10% w/w PEG droplet was placed on the target mark. The centers of the target mark and the droplet were determined by fitting circles into them. The circles were uniquely defined by selecting three points on each of them. The positioning error was calculated by measuring the difference between the centers of the target mark and the droplet. B) Positioning error of shrimp embryo-laden droplets (PS: printing system and MVS: machine vision system) (*n* = 7). The measurement resolution, based on the single pixel size, was ca. 15 µm.


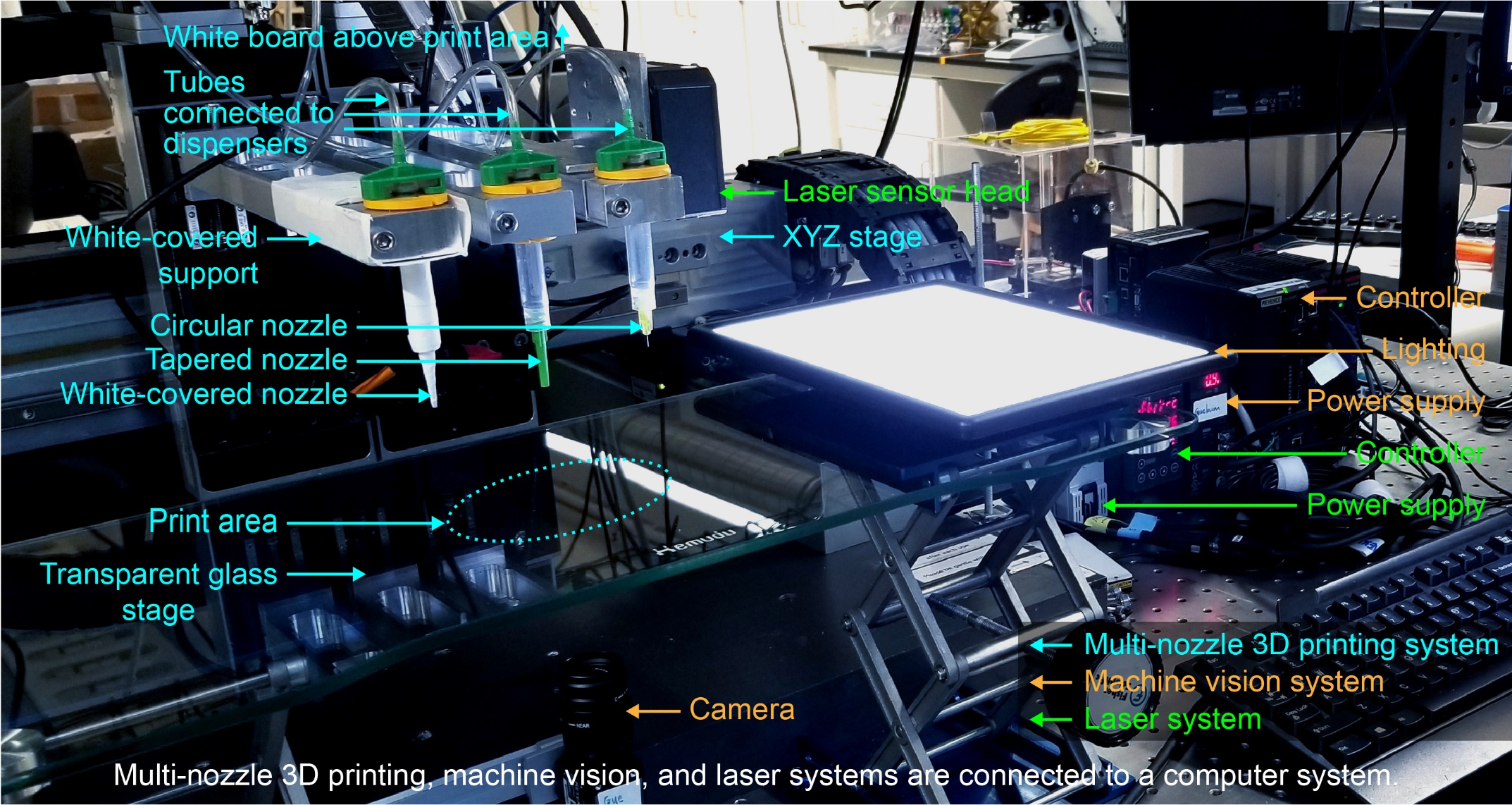


Figure S13. Aspiration-assisted adaptive printing system developed for beetles. A multi-nozzle printing process guided with machine vision and laser systems adapted to real-time spatial and visual data of moving beetles and target substrates in a closed-loop manner. The real-time data was acquired by a vision camera underneath a transparent glass stage. For enhanced detection of beetles, the white-covered nozzle, support, and the white board were used to reduce image background noise and increase the contrast between the background and beetles.


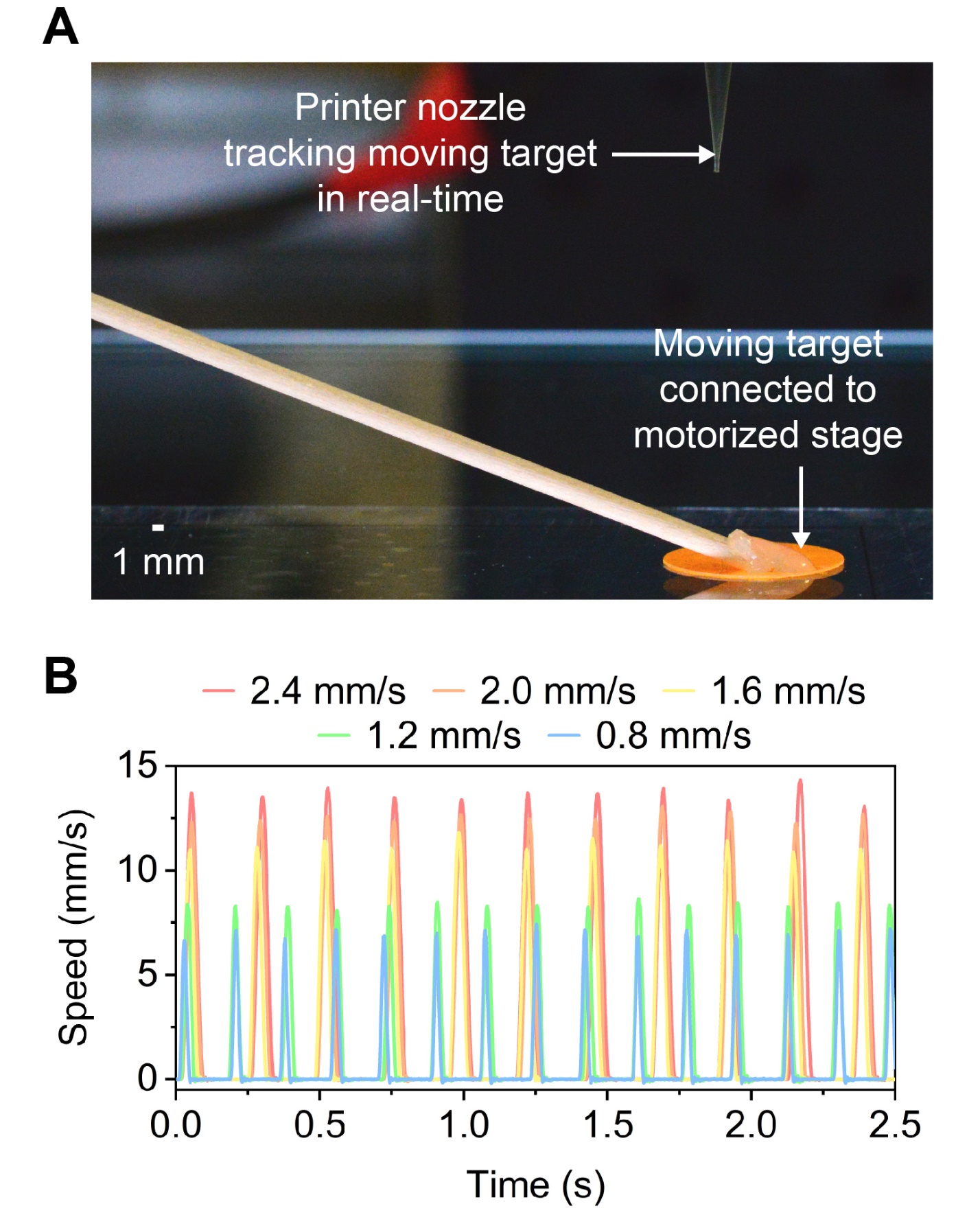


Figure S14. Tracking performance of the machine vision-guided adaptive printing system for moving targets. A) Image of experimental setup. The adaptive printing system tracked a circular target that was moved at speeds of 0.8, 1.2, 1.6, 2.0, and 2.4 mm/s by a motorized stage. A 200 µm diameter nozzle (7018417, Nordson EFD) tracked the moving target using its real-time location detected by the pattern search function of the machine vision system (detection conditions: search sensitivity = ca. 86%, accuracy = 100%, and minimum match percentage = 15%) (Table S2). B) Stage speeds of the adaptive printing system while tracking a circular target object that is moving at different speeds.


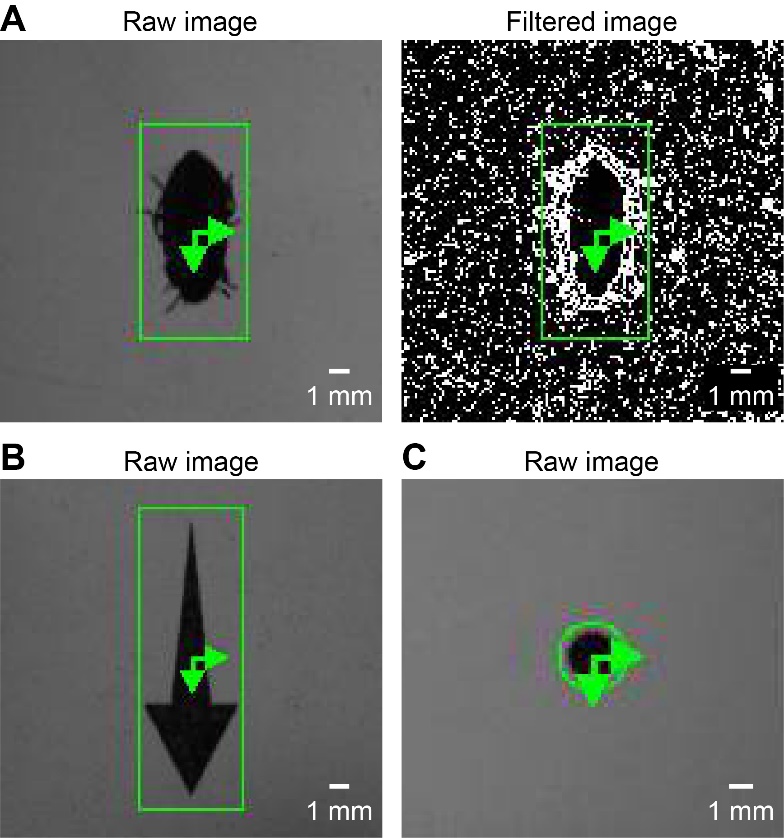


Figure S15. Detection of beetles and substrates using the machine vision system. A) Raw and filtered images of a beetle from the machine vision system. To detect moving beetles, three filters were applied to raw images captured by a vision camera: Sobel (count = 3), contrast conversion (offset = -73 and span = 2), and binary (upper limit = 255 and lower limit = 29) filters. These filters enhanced the visibility of the outlines of the beetles while eliminating extraneous objects in the background, such as a nozzle and mechanical support. A pattern search function (detection conditions: search sensitivity = ca. 86%, accuracy = 100%, and minimum match percentage = 15%) was applied to filtered images for real-time detection of beetles. Raw images of target B) arrow and C) circle from machine vision system. A pattern search function (detection conditions: search sensitivity = 100%, accuracy = 100%, and minimum match percentage = 10%) was used to detect them based on their raw images. More detailed information about the filters and pattern search function can be found in Supporting Note S3 and Table S2.


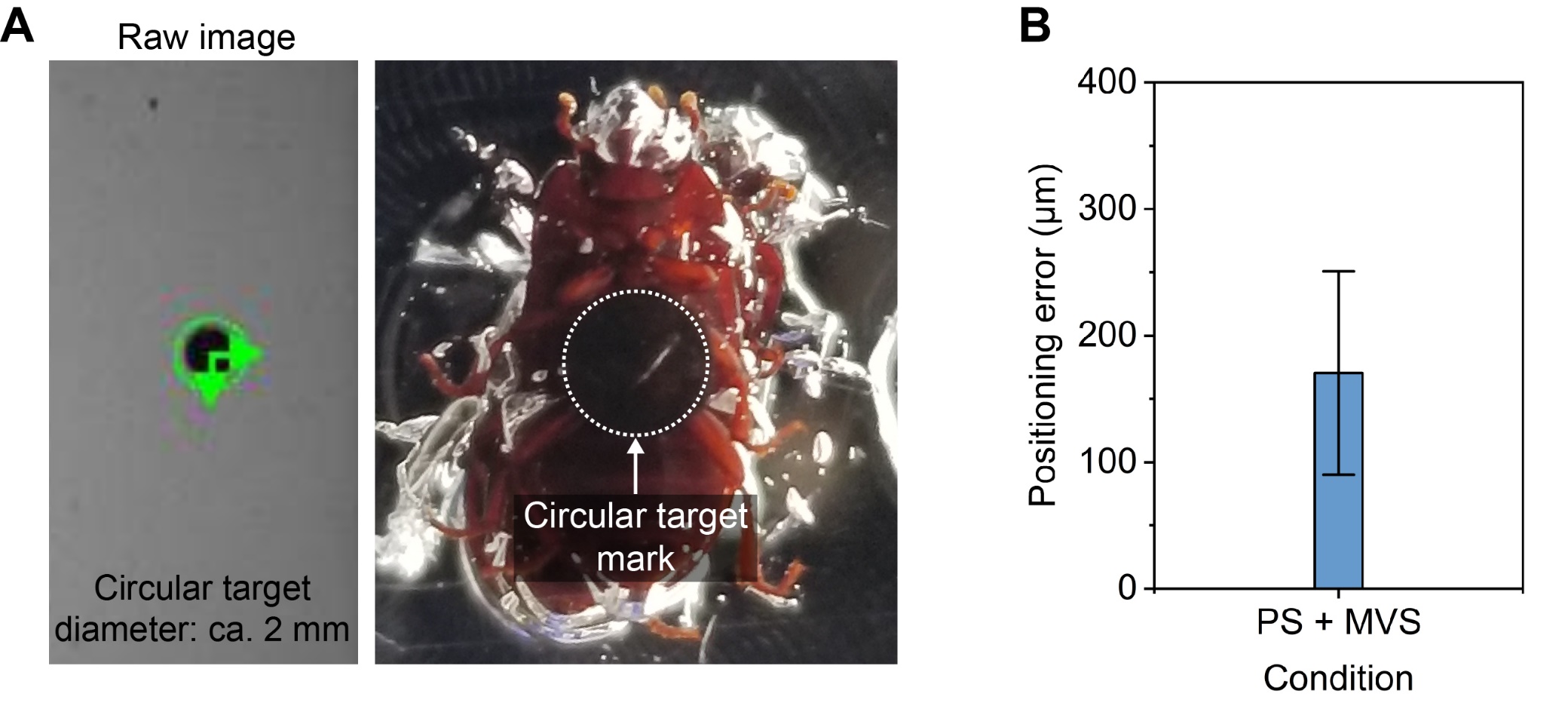


Figure S16. Estimation of the positioning error of the machine vision-guided adaptive printing system for beetles. A) Images showing a circular target (left) and beetle placed on top (right). B) Positioning error of beetles (PS: printing system and MVS: machine vision system) (*n* = 4). The positioning error was calculated by measuring the distance between the centers of a circular target and a beetle. The circular target was detected using the pattern search function of the machine vision system (detection conditions: search sensitivity = 100%, accuracy = 100%, and minimum match percentage = 10%). More detailed information about the pattern search function can be found in Table S2. The measurement resolution estimated from the single pixel size was ca. 10 µm.


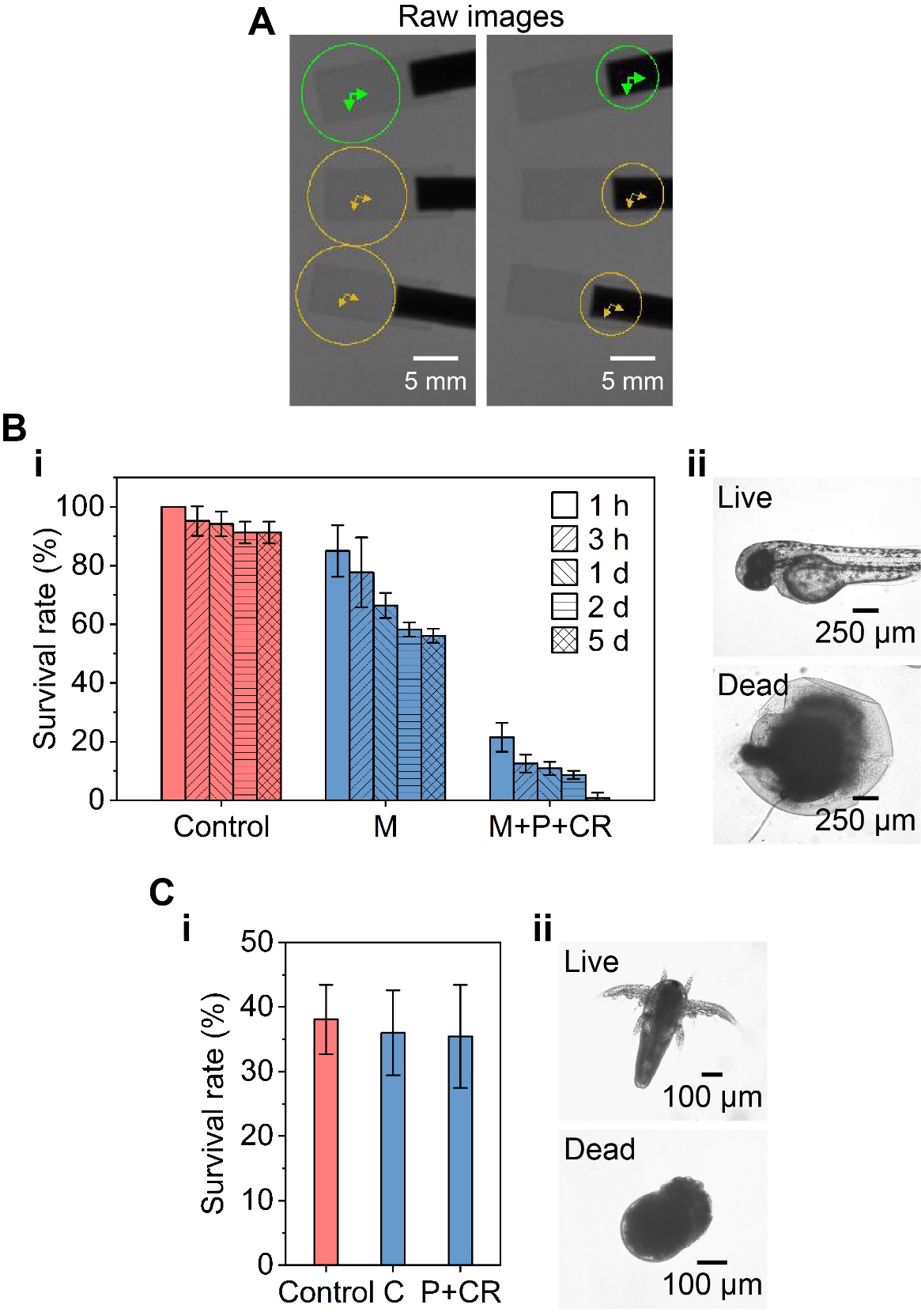


Figure S17. Cryopreservation and rewarming of the printed zebrafish and shrimp embryos. A) Raw images of cryotop devices from machine vision system. The machine vision system detected the locations of (left) multiple cryotop tips (transparent PP films) and (right) their bodies (wooden rods). The locations of the bodies were used to guide the laser system to measure their heights and calculate the heights of the transparent tips. Due to the transparency of the tips, their heights could not be directly measured by the laser system. These measurements provided the 3D locations of the cryotop tips. This enabled the adaptive printing (pick-and-place) of zebrafish embryo-included and shrimp embryo-laden cryoprotectant droplets on the cryotop tips. The pattern search function of the machine vision system was used to detect the cryotop tips and bodies (detection conditions: search sensitivity = ca. 71%, accuracy = ca. 67%, and minimum match percentage = 10%) (Table S2). B) Cryopreservation and laser rewarming of zebrafish embryo-included cryoprotectant droplets placed on cryotop tips: (i) survival rates of cryopreserved and rewarmed embryos based on criteria shown in Table S1 (M: microinjection of GNR-included cryoprotectant into embryos, P: printing process, and CR: cooling and rewarming) and (ii) optical microscope images of live (5 d) and dead (1 h) embryos after cryopreservation and rewarming. In (i), experiments were replicated 5 times with at least n = 15 embryos per trial. The results were consistent with manual manipulation ^[3,4]^. C) Cryopreservation and convective rewarming of shrimp embryo-laden cryoprotectant droplets placed on cryotop tips: (i) survival rates of cryopreserved and rewarmed embryos based on their hatch rates after 1d (C: cryoprotectant) (no significant difference between control and experimental groups: p ≥ 0.28) (n = 15) and (ii) optical microscope images of live and dead embryos 1 d after cryopreservation and rewarming. The control groups in B) and C) were not subjected to the pick-and-place printing processes, cryopreservation, and rewarming processes.

**
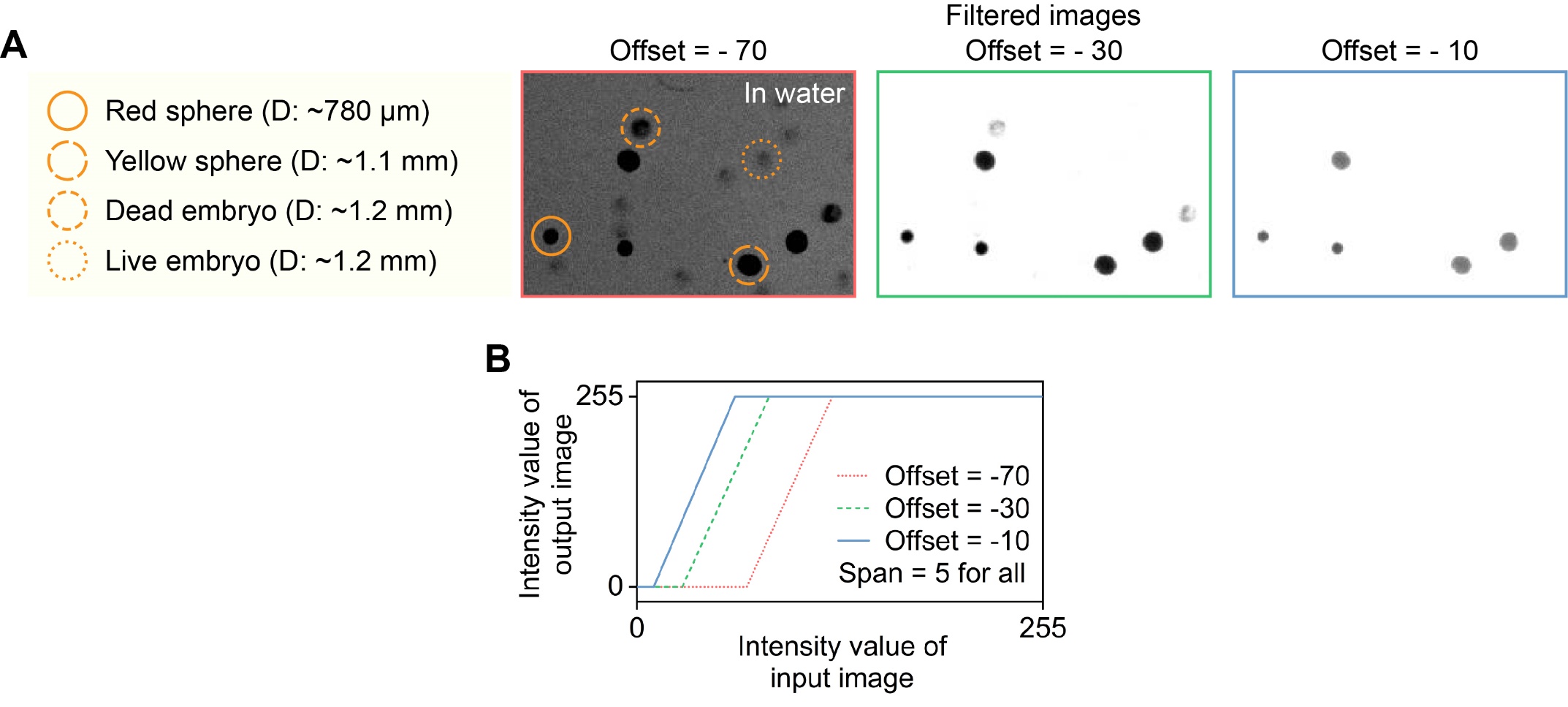
**

**Figure S18.** Filtered images used to sort live zebrafish embryos from microspheres and dead zebrafish embryos using the aspiration-assisted adaptive printing system. A) Images processed with contrast conversion filters (D: diameter) and B) their functions. In the machine vision system, the different contrast conversion filters were used to enhance the detection of live embryos by eliminating microspheres and dead embryos. At a fixed offset value, the span value determined the range of tonal values for contrast modification. The locations of the embryos and microspheres were identified using the pattern search function (detection conditions: search sensitivity = 100%, accuracy = 100%, and minimum match percentage = 10%). More information about the filter and search function is given in Supporting Note S3 and Table S2.

**
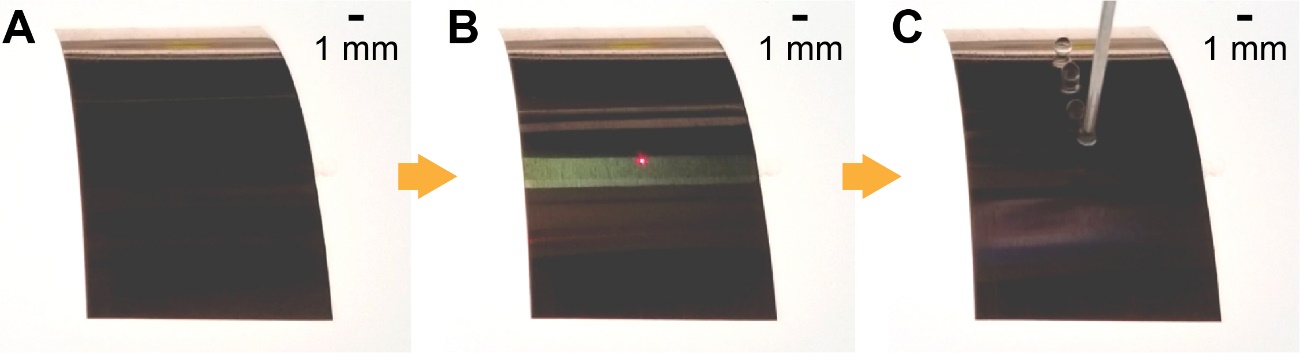
**

**Figure S19.** Steps to place zebrafish embryos on a curved surface. A) The machine vision system detected the location of a curved copper surface, B) the laser system measured the curved surface profile, and C) the printing system picked and placed zebrafish embryos on the curved surface. The location of the curved surface was detected using the pattern search function of the machine vision system (detection conditions: search sensitivity = ca. 71%, accuracy = ca. 67%, and minimum match percentage = 10%) (Table S2).


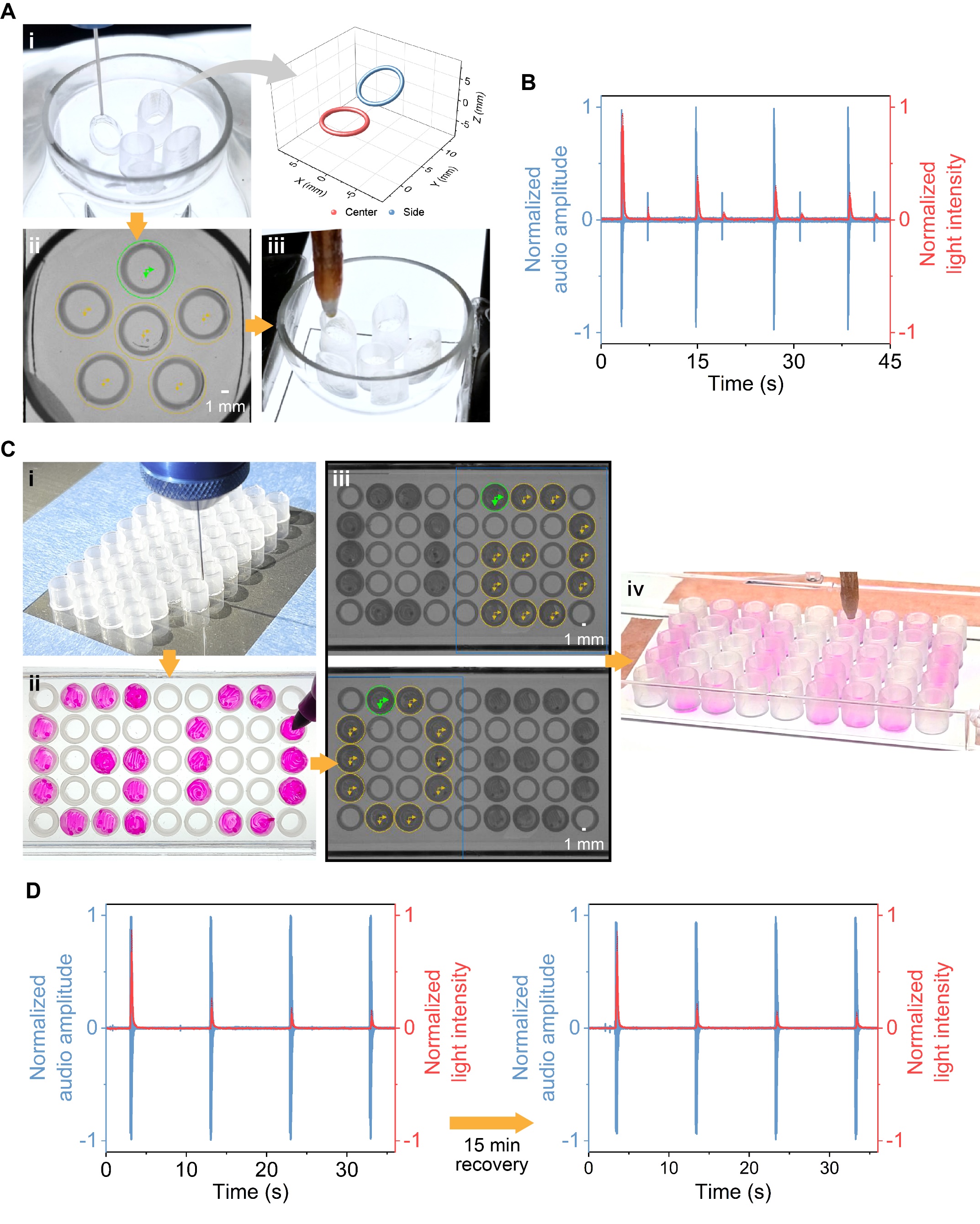


**Figure S20.** Printing process and functional testing of the dinoflagellate-based displays. A) Printing a dinoflagellate-based spherical display: (i) conformal printing of silicone chambers on a glass hemisphere (left) based on prescribed profiles (right), (ii) raw image from the machine vision system showing the detection of silicone chamber locations, and (iii) printing dinoflagellates in seawater into the chambers. The locations of the silicone chambers were detected by using the pattern search function of the machine vision system (detection conditions: search sensitivity = 100%, accuracy = 100%, and minimum match percentage = 0%) (Table S2). The height of the central chamber was measured for printing by the laser system. B) Result of functional testing of the dinoflagellate-based spherical display. Mechanical vibrations, induced by high- and low-amplitude audio tracks, were applied to the spherical display. The pixel-like chambers, which were loaded with dinoflagellates, exhibited bioluminescence in response to the vibrations. The intensity of the emitted light was dependent on both the amplitude of the audio and the number of repetitions. C) Printing a dinoflagellate-based planar display: (i) printing of silicone chambers on a PET film, (ii) hand-drawn circles to write the letters “GO”, (iii) raw image from a machine vision system showing the detection of the chambers with hand-drawn circles, and (iv) printing dinoflagellates in seawater into the chambers. The locations of the silicone chambers with the hand-drawn circles were detected by using the pattern search function of the machine vision system (detection conditions: search sensitivity = ca. 71%, accuracy = ca. 67%, and minimum match percentage = 15%) (Table S2). The height of the top left chamber was measured for printing dinoflagellates using the laser system. The hand-drawn circles were removed with 70% ethanol. D) Functional testing of the dinoflagellate-based planar display. To investigate the recovery of the light in the planar display, two vibrations induced by audio were applied at an interval of 15 min. The chambers emitted bioluminescence when subjected to audio-induced vibrations. The intensity of the emitted light was restored after the recovery time.

**
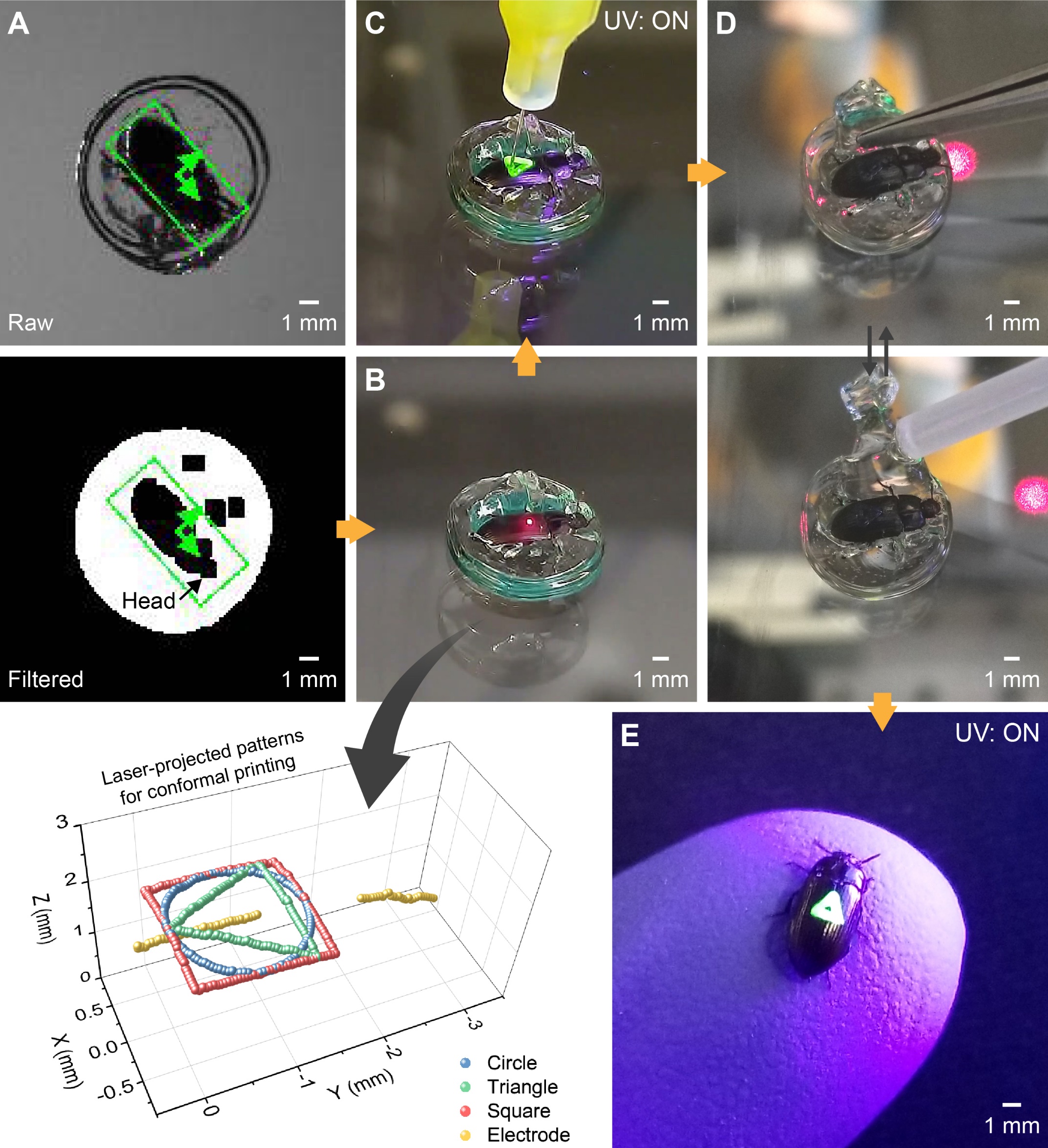
**

**Figure S21.** Steps to integrate customizable symbols and electrodes with beetles. A) Raw and filtered images showing the detection of the location and angle of a beetle placed in a Pluronic hydrogel structure using the machine vision system. Sobel (count = 2), dark noise removal (processing shape = square, size = 3, direction = XY, and count = 3), and binary (upper limit = 255 and lower limit = 230) filters were applied to the raw image to highlight the beetle body placed in the hydrogel for detection. The pattern search function (detection conditions: search sensitivity = ca. 14%, accuracy = 100%, and minimum match percentage = 10%) was applied to the filtered image to detect the location and angle of the beetle (Supporting Note S3 and Table S2). B) Laser scanning to project a two-dimensional symbol on the beetle. C) Conformal printing of a symbol (3 layers) on a beetle by using its projected toolpath. D) Removal of Pluronic hydrogel with tweezers and water (repeated). E) Live beetle with secret triangular symbol. UV light was illuminated in C) and E) to display the UV-responsive secret symbol. This printing process was also used to integrate electrodes with the beetles.


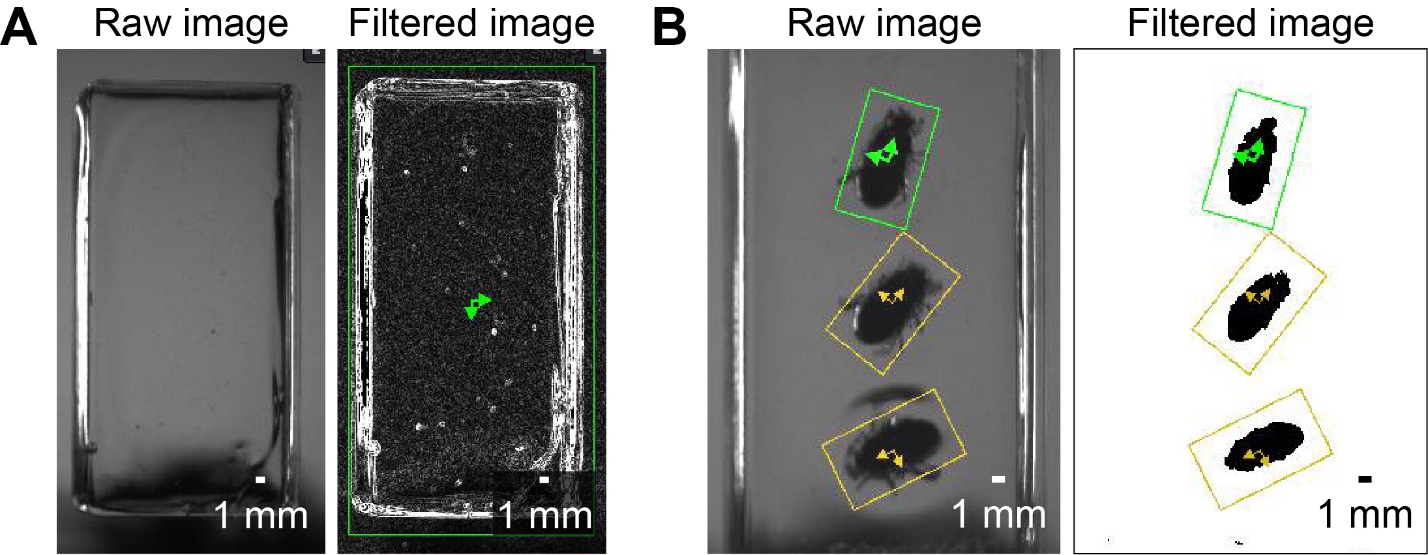


Figure S22. Using a machine vision detection system to place the beetles in a hydrogel bath and print electrodes on them. A) Raw and filtered images of the Pluronic hydrogel bath from the machine vision system. The machine vision system used the Sobel filter (count = 2) and the pattern search function (detection conditions: search sensitivity = 0%, accuracy = 50%, and minimum match percentage = 0%) to detect the location and angle of the bath in order to place beetles in it. B) Raw and filtered images of multiple beetles placed in the Pluronic hydrogel bath from the machine vision system. The machine vision system used the binary filter (upper limit = 255 and lower limit = 11) and the pattern search function (detection conditions: search sensitivity = 100%, accuracy = 100%, and minimum match percentage = 15%) to detect the locations and angles of the beetles. The spatial information was used for the adaptive printing of electrodes on beetles. Supporting Note S3 and Table S2 provide additional information about the filters and search function.


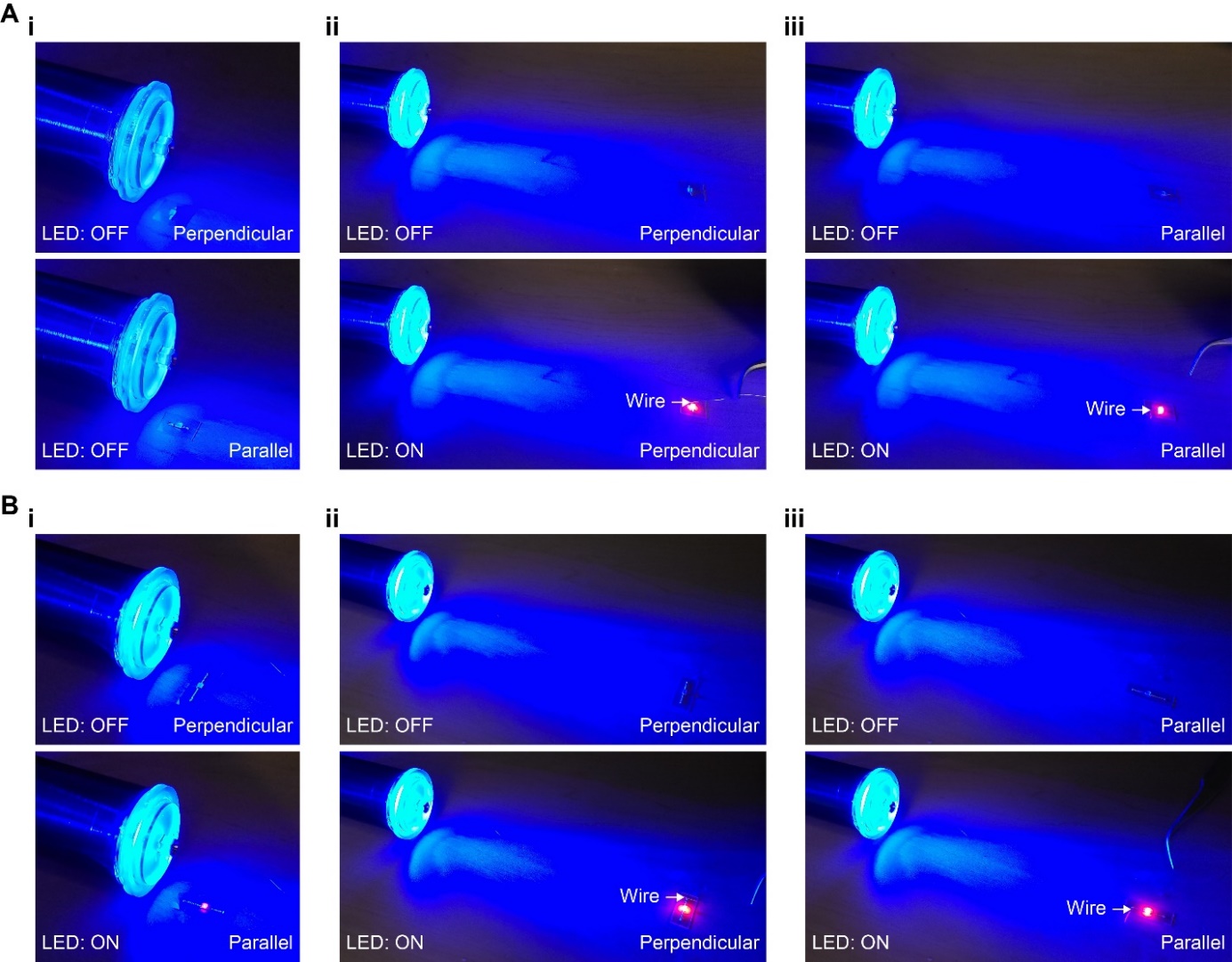


Figure S23. Functional testing of the LEDs via wireless power transmission. A) LED integrated with printed short silver electrodes (length: ca. 4 mm). (i) The LED was not turned on regardless of its direction relative to the Tesla coil column placed at a close distance (10 - 25 mm). When the Tesla coil was extremely close to the LED (1 - 3 mm), it was turned on but rapidly damaged due to electrical arcing from the Tesla coil. (ii and iii) When an extended wire was used to enhance the wireless power transmission, the LED was turned on in both perpendicular and parallel directions relative to the Tesla coil placed at a far distance (70 - 100 mm). B) LED integrated with printed long silver electrodes (length: ca. 12 mm). (i) The LED was turned on when it was parallel to the axis of the Tesla coil column at a close distance (10 - 25 mm). (ii and iii) The wireless power transmission was improved by using an extended wire. The LED was then turned on in perpendicular and parallel directions relative to the Tesla coil column placed at a far distance (70 - 100 mm). These results suggested that (1) the wireless power transmission from the Tesla coil can be improved with the extended wire, and (2) optimized electrode designs can allow the LED to be powered by the Tesla coil without the extended wire.

Table S1. Survival criteria of zebrafish embryos.

| Time | Survival criterion adopted from previous studies ^[4,8]^ |
| --- | --- |
| 1 d | Movement and heartbeats |
| 2 d | Eye pigmentation |
| $\geq$ 3 d | Swimming, straight trunk musculature, and the presence of a normal heart |

**Table S2.** Image processing filters and pattern search function of the machine vision system.

| Type | | Effect (●) and parameter (○) | Example  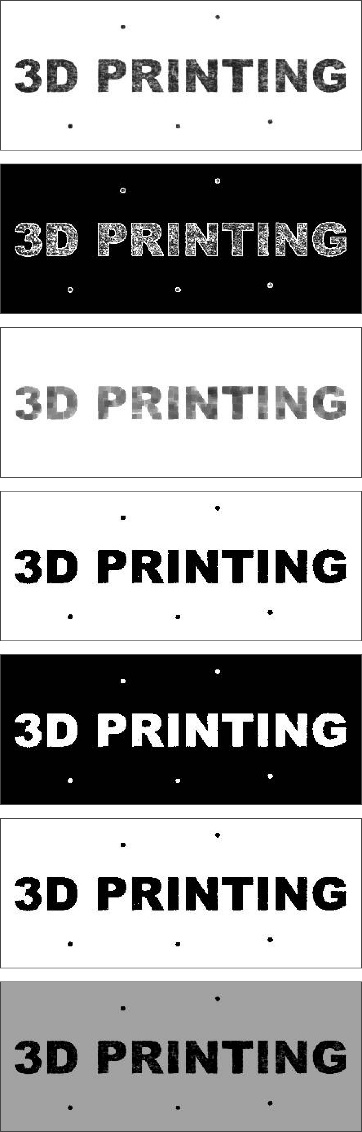  Original image |
| --- | --- | --- | --- |
| Filter | Contrast conversion | - This controls the span and offset functions to highlight the contrast of particular tones of an image. - Offset: Higher values make images lighter. - Span: Higher values make the tone rich. | 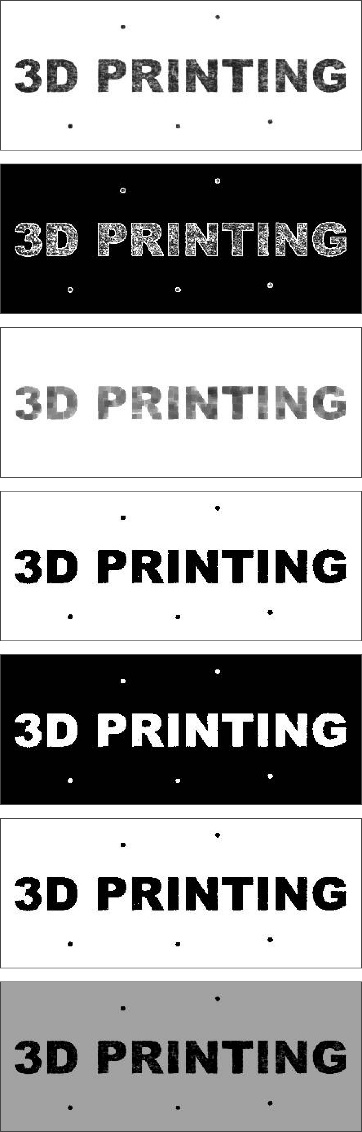 |
|  | Sobel | - This extracts and merges the edges of an image in X and Y directions to emphasize them. - Count: This controls the applicable count of the filter. | 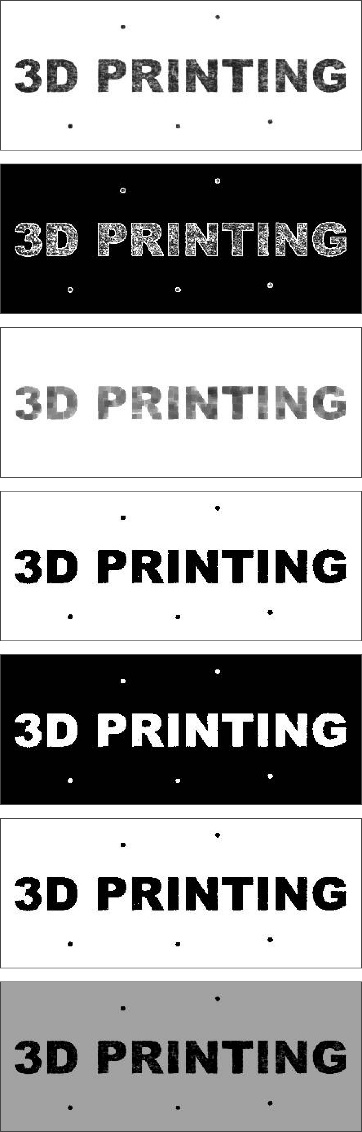 |
|  | Binary | - This binarizes an image by converting pixels in a particular intensity range to white and other pixels to black. The upper and lower limit threshold values define the range of intensity. - Upper limit: This sets an upper intensity limit. - Lower limit: This sets a lower intensity limit. | 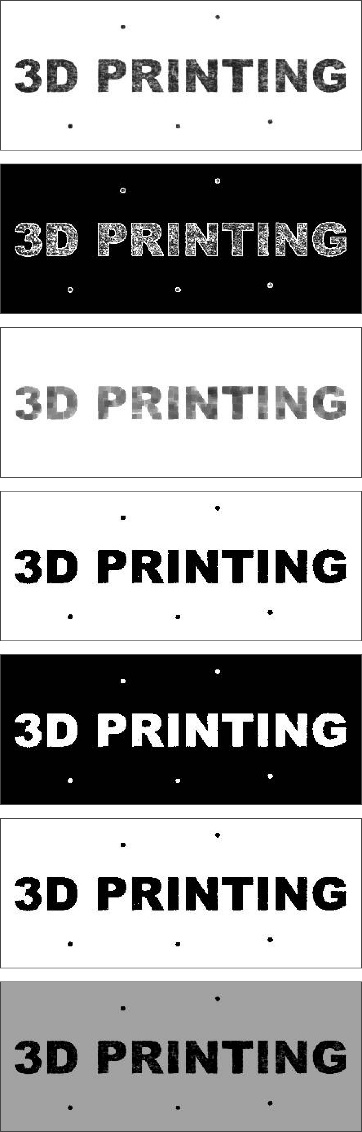 |
|  | Dark noise removal | - This eliminates the dark noise of an image. - Processing shape: This defines the processing shape of the filter. - Size: This defines the reference pixels of an image for enhancement. - Direction: This defines the processing direction of the filter. - Count: This defines the number of filter processes. | 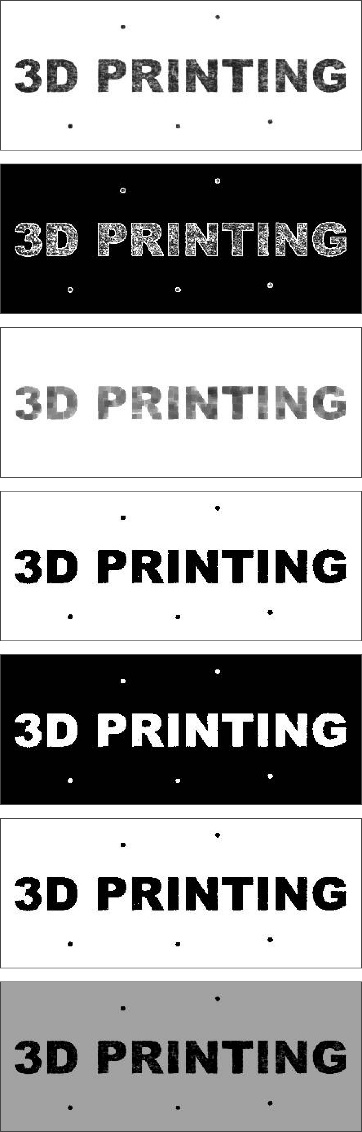 |
| Function | Pattern search | - This discerns the positions and angles of one or more patterns resembling a reference pattern within an image. - Search sensitivity: This controls the compression ratio of a reference pattern. - Accuracy: This controls the number of times the search processes are repeated. - Minimum match: This defines a cut-off threshold correlation value about the similarity between the reference pattern and pattern searched in an image. | - |

Table S3. Evaluation of the mesh filters for organisms. Mesh-filtered nozzles were constructed by combining 1200 μm diameter nozzles with nylon filters with mesh sizes of 18 μm, 60 μm, and 250 μm. The performance of the mesh-filtered nozzles was evaluated by picking up dinoflagellates, shrimp embryos, and shrimp larvae in seawater under vacuum pressure (4 kPa). Nylon filters with mesh sizes of 18 μm and 60 μm successfully filtered organisms from seawater, while a nylon filter with a mesh size of 250 μm was unsuccessful.

| Sample  Mesh size | Dinoflagellates | Shrimp embryos | Shrimp larvae |
| --- | --- | --- | --- |
| 18 μm | O | O | O |
| 60 μm | O | O | O |
| 250 μm | X | X | X |

Table S4. Printing conditions used for the experiments.

| Material | Immersion solution | Tip diameter (μm) | Mesh size  (μm) | Vacuum pressure  (kPa) | Positive pressure  (psi) | Speed  (mm/s) |
| --- | --- | --- | --- | --- | --- | --- |
| Zebrafish embryos (Figure 1) | Freshwater | 510  (8001089, Fisnar) | N/A | 2.5 | 0 | N/A |
| Dinoflagellates (Figure 2) | Seawater | 250  **(**8001274, Fisnar) | 18  **(**ME 17233, Tisch Scientific) | 2.5  for 0.1 s - 3 s | 0.7  for 0.01 s - 0.3 s | N/A |
|  |  | 580  **(**8001272, Fisnar) | 18  **(**ME 17233, Tisch Scientific) | 2.5  for 0.1 s - 3 s | 0.7  for 0.01 s - 0.3 s | N/A |
|  |  | 1200  **(**8001270, Fisnar) | 18  **(**ME 17233, Tisch Scientific) | 2.5  for 0.1 s - 3 s | 0.7  for 0.01 s - 0.3 s | N/A |
| Shrimp embryos (Figure 2) | Seawater | 250  **(**8001274, Fisnar) | 18  **(**ME 17233, Tisch Scientific) | 4  for 0.1 s - 0.9 s | 0.7  for 0.03 s | N/A |
|  | 10% PEG | 410  (8001273, Fisnar) | 60  **(**ME 17228, Tisch Scientific) | 2  for 0.5 s - 7 s | 1  for 0.03 s | N/A |
|  | 20% PEG | 410  (8001273, Fisnar) | 60  **(**ME 17228, Tisch Scientific) | 4  for 1 s - 14 s | 1.3  for 0.03 s | N/A |
| Shrimp larvae  (Figure 2) | Seawater | 580  **(**8001272, Fisnar) | 18  **(**ME 17233, Tisch Scientific) | 4  for 0.2 s | 0.7  for 0.03 s | N/A |
|  | Seawater | 840  (8001271, Fisnar) | 18  **(**ME 17233, Tisch Scientific) | 4  for 0.2 s | 0.7  for 0.03 s | N/A |
|  | Seawater | 1200  (8001270, Fisnar) | 18  **(**ME 17233, Tisch Scientific) | 4  for 0.2 s | 0.7  for 0.03 s | N/A |
|  | 10% PEG | 580  **(**8001272, Fisnar) | 60  **(**ME 17228, Tisch Scientific) | 2  for 1 s | 1  for 0.03 s | N/A |
|  | 10% PEG | 840  (8001271, Fisnar) | 60  **(**ME 17228, Tisch Scientific) | 2  for 1 s | 1  for 0.03 s | N/A |
|  | 10% PEG | 1200  (8001270, Fisnar) | 60  **(**ME 17228, Tisch Scientific) | 2  for 1 s | 1  for 0.03 s | N/A |
|  | 20% PEG | 580  **(**8001272, Fisnar) | 60  **(**ME 17228, Tisch Scientific) | 4  for 2 s | 1.3  for 0.03 s | N/A |
|  | 20% PEG | 840  (8001271, Fisnar) | 60  **(**ME 17228, Tisch Scientific) | 4  for 2 s | 1.3  for 0.03 s | N/A |
|  | 20% PEG | 1200  (8001270, Fisnar) | 60  (ME 17228, Tisch Scientific) | 4  for 2 s | 1.3  for 0.03 s | N/A |
| Auxiliary nozzle (Figure 2) | N/A | 1370  (8001292, Fisnar) | N/A | 4 | N/A | N/A |
| Stirring nozzle  (Figure 2) | N/A | 840  (8001107, Fisnar) | N/A | N/A | 2  for 0.4 s | N/A |
| Beetles  (Figure 3) | N/A | 1200  (8001270, Fisnar) | N/A | 4 | 0 | N/A |
| Pluronic hydrogel for fixing beetles  (Figure 3) | N/A | 3000  (8001217, Fisnar) | N/A | N/A | 9 | 10 |
| Zebrafish embryos for cryopreservation (Figure 4) | Freshwater | 510  (8001089, Fisnar) | N/A | 2.5 | 0 | N/A |
| Cryoprotectant agent for zebrafish embryos (Figure 4) | N/A | 150  (7018424, Nordson EFD) | N/A | N/A | 8  for 0.25 s | N/A |
| Shrimp embryos for cryopreservation (Figure 4) | Cryoprotectant agent | 250  **(**8001274, Fisnar) | 60  (ME 17228, Tisch Scientific) | 4 for 2 s | 1.3  for 0.03 s | N/A |
| Sorting and conformal printing of zebrafish embryos (Figure 4) | Freshwater | 510  (8001089, Fisnar) | N/A | Zebrafish embryos: 2.5    Microspheres: 4.0 | 0 | N/A |
| Silicone chambers for displays  (Figure 4) | N/A | 330  (7018305, Nordson EFD) | N/A | N/A | 280 | 2 |
| Dinoflagellates for displays (Figure 4) | N/A | 1200  (8001270, Fisnar) | 18  (ME 17233, Tisch Scientific) | 2.5 for 3 s | 0.7  for 0.1 s | N/A |
| Pluronic hydrogel for symbols  (Figure 4) | N/A | 100  (7018462, Nordson EFD) | N/A | N/A | 70 | 1 |
| Silver epoxy adhesive for electrodes  (Figure 4) | N/A | 150  (7018424, Nordson EFD) | N/A | N/A | 90 | 1 |
| LED (Figure 4) | N/A | 510  (8001089, Fisnar) | N/A | 4 | 0 | N/A |

- After placing organisms (zebrafish embryos, dinoflagellates, shrimp embryos, and shrimp larvae), the residual solutions in the nozzles were cleaned by alternately applying positive (4-5 psi for 0.3-1 s) and vacuum (4 kPa for 0.3-1 s) pressures every cycle.
- The travel speed of the printing system was set to its maximum limit of 2 m/s. It attempted to reach this speed while in motion.
- For the Pluronic hydrogel for symbols, silicone chambers for displays, and silver epoxy adhesive for electrodes, the distance between the end of the nozzle and the substrate for printing the first layer was 60-70% of the nozzle diameter.

Table S5. Repeatedly used solutions for the experiments.

| Solution | Component |
| --- | --- |
| Freshwater for incubation of zebrafish embryos | - Freshwater was formulated by mixing sea salt (SS3-50, Instant Ocean) with deionized water at a concentration of 0.06 % w/v. |
| Seawater for incubation of dinoflagellates | - Seawater with a salinity of 32 ppt was made as needed by mixing sea salt (SS3-50, Instant Ocean) with deionized water. In all experiments, the salinity of seawater was measured using a refractometer (Xindacheng). |
| Seawater for incubation of shrimp embryos and larvae | - Seawater with a salinity of 32 ppt was created by combining sea salt (SS3-50, Instant Ocean) with deionized water. |
| Pluronic hydrogel used for (1) printing dots to measure tracking errors of adaptive printing system for beetles, (2) fixing beetles in desired locations, and (3) printing symbols on beetles | - A 10% v/v solution was created by combining glycerol (Sigma-Aldrich) and deionized water. Pluronic hydrogel was created by adding Pluronic 127 (Sigma-Aldrich) to the solution at a concentration of 40% w/v. |
| Solutions used for cryopreservation of  zebrafish embryo | - The cryoprotectant agent A consisted of 13.1 M propylene glycol (Sigma-Aldrich) and 9.4 $\times$ 10^17^ np/m^3^ GNRs (plasmonic 1064 nm, nanoComposix) in freshwater ^[4]^. - The cryoprotectant agent B consisted of 2 M propylene glycol (Sigma-Aldrich), 1.2 M methanol (Sigma-Aldrich), 0.5 M trehalose dihydrate (Sigma-Aldrich), and 4.2 $\times$ 10^17^ np/m^3^ GNRs (plasmonic 1064 nm, nanoComposix) in freshwater ^[4]^. - The post-warming bath consisted of 1 M propylene glycol (Sigma-Aldrich), 0.6 M methanol (Sigma-Aldrich), and 0.25 M trehalose dihydrate (Sigma-Aldrich) in freshwater ^[4]^. - Freshwater used in the above solutions was created by mixing sea salt (SS3-50, Instant Ocean) with deionized water at a concentration of 0.06% w/v. |
| Solutions used for  cryopreservation of  shrimp embryo | - The cryoprotectant agent consisted of 7.5% w/w propylene glycol (Sigma-Aldrich), 15% w/w methanol (Sigma-Aldrich), and 20% w/w PEG (Alfa Aesar) in seawater. - Seawater in the above solution was made by dissolving sea salt (SS3-50, Instant Ocean) in deionized water at a salinity of 32 ppt. |

Movie S1. Picking and placing zebrafish embryos.

Movie S2. Picking and placing shrimp embryo-laden droplets.

Movie S3. Tracking and picking beetles.

Movie S4. Picking and placing freely moving beetles.

Movie S5. Placing organisms on cryotop devices for cryopreservation.

Movie S6. Sorting and conformal placing of zebrafish embryos.

Movie S7. Dinoflagellate-based displays.

Movie S8. Beetles with integrated symbols.

Movie S9. Beetles with integrated LEDs.

**References**

[1] R. K. Korhonen, M. S. Laasanen, J. Töyräs, J. Rieppo, J. Hirvonen, H. J. Helminen, J. S. Jurvelin, *J. Biomech.* **2002**, *35*, 903.

[2] Deok-Ho Kim, Chang Nam Hwang, Yu Sun, Sang Ho Lee, Byungkyu Kim, B. J. Nelson, *IEEE Trans. Nanobioscience* **2006**, *5*, 89.

[3] K. Khosla, Y. Wang, M. Hagedorn, Z. Qin, J. Bischof, *ACS Nano* **2017**, *11*, 7869.

[4] K. Khosla, J. Kangas, Y. Liu, L. Zhan, J. Daly, M. Hagedorn, J. Bischof, *Adv. Biosyst.* **2020**, *4*, 2000138.

[5] Y. Tomizawa, K. Dixit, D. Daggett, K. Hoshino, *Sensors* **2019**, *19*, 1506.

[6] B. Tesson, M. I. Latz, *Biophys. J.* **2015**, *108*, 1341.

[7] J. Lomakin, P. A. Huber, C. Eichler, Y. Arakane, K. J. Kramer, R. W. Beeman, M. R. Kanost, S. H. Gehrke, *Biomacromolecules* **2011**, *12*, 321.

[8] M. Janik, F. W. Kleinhans, M. Hagedorn, *Cryobiology* **2000**, *41*, 25.
